# Genetic variation in long noncoding RNAs and the risk of nonalcoholic fatty liver disease

**DOI:** 10.18632/oncotarget.15286

**Published:** 2017-02-11

**Authors:** Silvia Sookoian, Cristian Rohr, Adrián Salatino, Hernán Dopazo, Tomas Fernandez Gianotti, Gustavo O. Castaño, Carlos J. Pirola

**Affiliations:** ^1^ Department of Clinical and Molecular Hepatology, Institute of Medical Research A Lanari-IDIM, University of Buenos Aires – National Scientific and Technical Research Council (CONICET), Ciudad Autónoma de Buenos Aires, Argentina; ^2^ Biomedical Genomics and Evolution Laboratory, Ecology, Genetics and Evolution Department, Faculty of Science, IEGEBA, University of Buenos Aires, National Scientific and Technical Research Council (CONICET), Ciudad Autónoma de Buenos Aires, Argentina; ^3^ Department of Molecular Genetics and Biology of Complex Diseases, Institute of Medical Research A Lanari-IDIM, University of Buenos Aires, National Scientific and Technical Research Council (CONICET), Ciudad Autónoma de Buenos Aires, Argentina; ^4^ Liver Unit, Medicine and Surgery Department, Hospital Abel Zubizarreta, Ciudad Autónoma de Buenos Aires, Argentina

**Keywords:** NAFLD, gene expression, lncRNAs, nonalcoholic steatohepatitis, epigenetics

## Abstract

The human transcriptome comprises a myriad of non protein-coding RNA species, including long noncoding RNAs (lncRNAs), which have a remarkable role in transcriptional and epigenetic regulation. We hypothesized that variants in lncRNAs influence the susceptibility to nonalcoholic fatty liver disease (NAFLD). Using next generation sequencing, we performed a survey of genetic variation associated with randomly selected lncRNA-genomic regions located within both experimentally validated and computationally predicted regulatory elements. We used a two-stage (exploratory, *n* = 96 and replication, *n* = 390) case-control approach that included well-characterized patients with NAFLD diagnosed by liver biopsy. We sequenced > 263 megabase pairs at quality score > Q17, in a total of 2,027,565 reads, including 170 lncRNA-genomic regions. In the sequencing analysis and the validated dataset, we found that the rs2829145 A/G located in a lncRNA (lnc-JAM2-6) was associated with NAFLD and the disease severity. Prediction of regulatory elements in lnc-JAM2-6 showed potential sequence-specific binding motifs of oncogenes *MAFK* and *JUND*, and the transcription factor *CEBPB* that is involved in inflammatory response. The A-allele was significantly associated with NAFLD as disease trait (*p* = 0.0081) and the disease severity (NASH-nonalcoholic steatohepatitis vs. controls: OR 2.36 [95% CI: 1.54−3.62], *p* = 0.000078). The A-allele carriers also have significantly higher body mass index and glucose-related traits compared with homozygous GG. Hence, our results suggest that variation in lncRNAs contributes to NAFLD severity, while pointing toward the complexity of the genetic component of NAFLD, which involves still unexplored regulatory regions of the genome.

## INTRODUCTION

Nonalcoholic fatty liver disease (NAFLD) is the most frequent cause of chronic liver disease worldwide [1]. The natural history of the disease develops into disease stages that may progress from a relatively benign histological form characterized by plain fat accumulation (referred to as simple steatosis or nonalcoholic fatty liver -NAFL) to a more severe histological picture characterized by liver cell injury, a mixed inflammatory lobular infiltrate, and variable fibrosis known as nonalcoholic steatohepatitis (NASH) [2].

The pathogenesis of NAFLD implies a complex interaction of many factors, including genetic predisposition and environmental insults [3, 4]. In addition, epigenetic regulation of liver gene expression has also been demonstrated in human studies [5–9].

The genetic component of NAFLD has been extensively explored in the last decade by several strategies, including candidate-gene [3] and genome-wide association studies (GWAS) [10–15], which elucidated the role of genetics in the disease severity [16] and the interaction with cardiovascular disease [17]. Furthermore, epigenetic and genetic variation in the mitochondrial DNA has also been recently reported in association with the disease severity and progression [6,18]. Nevertheless, the variants identified thus far explain at most 10−20% of the disease heritability [14, 17, 15, 16, 19]. It is then reasonable to expect that variants that have not yet been identified explain the full spectrum of the genetic component of NAFLD, including variants located in regulatory regions of the genome. In fact, a large proportion (> 90%) of risk alleles uncovered by GWAS that are associated with the genetic component of complex diseases/traits reside in noncoding protein genes or long intergenic (lincRNA)/long noncoding (lncRNAs) RNA regions [20]. LncRNAs can be sense, antisense or bidirectional, and intronic or intergenic with respect to protein-coding loci [21, 22].

LncRNAs, which have been shown to cover a significant portion of the noncoding transcriptome in mammalian genomes, regulate critical aspects of the genome biology, including binding to chromatin and the assembly of ribonuclear protein complexes [21]. More importantly, lncRNAs orchestrate cellular strategies of trans-differentiation, organ regeneration and metabolic reprogramming, while also having tissue-specific expression patterns [21].

The role of genetic variation of lncRNA-genomic regions in the risk of NAFLD remains largely unexplored. In this study, we hypothesized that variants in lncRNA loci explain part of the genetic component associated with NAFLD susceptibility. Hence, as a proof of concept, we first performed a global survey of genetic variation associated with randomly selected lncRNA regions across the genome by utilizing next generation sequencing (NGS) technology. Further, we performed a subsequent association analysis of variants that merit replication in a larger independent sample.

## RESULTS

### Characterization of genetic variation in lncRNA-genomic regions by NGS

Clinical and biochemical features of patients and controls are shown in Table 1. In the exploratory study, we sequenced > 263 megabase pairs (Mb) at quality score > Q17, in a total of 2,027,565 reads of 140 base-pair (bp) fragments on average, including 170 lncRNA genomic regions encompassing 50 kb (annotation details of lncRNAs are provided in Supplementary Table 1. These regions were randomly selected across the genome to represent a portion of single nucleotide polymorphisms (SNPs) located in lncRNAs. Our findings revealed 93 SNPs, including 76 known variants and 17 that were novel or without annotation details in dbSNP. Full details on variants according to dbSNP, chromosome position, reference allele and intra study-minor allele-frequency (MAF) are disclosed in Supplementary Table 2.

**Table 1:** Exploratory study: Clinical and biochemical characteristics of control subjects and patients with NAFLD

Variables (mean ± SD)	Control subjects	NAFL	NASH
**Number of subjects**	32	32	32
**Age, years**	48.0 ± 7.4	51.9 ± 9.8	51.2 ± 11.0
**BMI, kg/m**^2^	23.0 ± 2.4	31.6 ± 4.7 *	36.0 ± 5.6 * ^ø^
**Waist circumference, cm**	80.0 ± 9.1	101.0 ± 8.6 *	112.0 ± 14.0 * ^ø^
**Fasting plasma glucose, mg/dL**	81.3 ± 7.2	96.5 ± 19.0 *	127.0 ± 49.0 * ^ø^
**Fasting plasma insulin, μU/mL**	5.3 ± 2.4	12.4 ± 6.5 *	20.9 ± 13.6 * ^ø^
**HOMA-IR index**	1.0 ± 0.5	3.0 ± 2.0 *	6.2 ± 4.6 * ^ø^
**SABP, mmHg**	116.0 ± 9.2	125.0 ± 11.0 *.5	133.0 ± 16.4 *
**DABP, mmHg**	72.5 ± 8.6	79.0 ± 8.0 *	79.0 ± 14.6 *
**Total cholesterol, mg/dL**	219 ± 51	208 ± 59	207 ± 46
**HDL-cholesterol, mg/dL**	57 ± 15	51 ± 29	47 ± 13
**LDL-cholesterol, mg/dL**	119 ± 36	127 ± 58	121 ± 42
**Triglycerides, mg/dL**	87 ± 30	149 ± 77 *	221 ± 142 *
**AST, U/L**	21.5 ± 4.4	38.0 ± 19.0 *	49.4 ± 33.0 *
**ALT, U/L**	21 ± 5	69 ± 101 *	60 ± 35 *
**GGT, U/L**	36 ± 26	48 ± 29.5	86 ± 82 * ^ø^
**AP, U/L**	160 ± 67	220 ± 89 *	269 ± 92 * ^ø^
**Degree of steatosis, %**	−	49 ± 23	60 ± 20 ^ø^
**Lobular inflammation (0–3)**	−	0.6 ± 0.5	1.4 ± 0.5 ^ø^
**Histological Features**			
**Portal inflammation (0–2)**	−	0	1.7 ± 0.7 ^ø^
**Hepatocellular ballooning (0–2)**	−	0	0.7 ± 0.6 ^ø^
**Fibrosis Stage**	−	0.1 ± 0.5	1.9 ± 1.2 ^ø^
**NAS**	−	2.4 ± 1.0	6.4 ± 1.6 ^ø^

Abbreviations: NAFL: nonalcoholic fatty liver, NASH: nonalcoholic steatohepatitis BMI: body mass index; SABP and DABP: systolic and diastolic arterial blood pressure; HOMA: homeostatic model assessment; ALT and AST: Serum alanine and aspartate aminotransferase; GGT: gamma-glutamyl-transferase; AP: alkaline phosphatase.

Results are expressed as mean ± SD. The *p* value pertains to the statistical significance calculated using Mann-Whitney *U* test. *Significant difference when compared with controls *p* < 0.05). ^ø^ Significant difference when compared with NAFL (*p* < 0.05).

The analysis of sequence data yielded two variants (rs2829145 and rs11171490) associated with NAFLD and related histological outcomes. Specifically, the rs2829145 located at AP000476.1 gene was significantly associated with ballooning degeneration (odds ratio -OR 2.89, 95% confidence interval -CI 1.06−7.86, *p* = 0.03), a histological feature associated with the disease progression [23]. The rs2829145 is a transcript variant in a novel lincRNA, annotated under the name lnc-JAM2-6 (http://www.lncipedia.org/db) or NONHSAG032538.2 (NONCODE database) (Supplementary Table 6). In addition, rs11171490 located at RP11-110A12.2 gene was significantly associated with NAFLD disease severity (based on the regression analysis for an ordinal multinomial distribution *p* = 0.005). The rs11171490 is a transcript variant that resides in a noncoding RNA annotated under the name lnc-OR6C70-1 (http://www.lncipedia.org/db) or NONHSAG011311.2 (NONCODE database) (Supplementary Table 6).

Complete features of rs2829145 and rs11171490, including their genomic location and gene names (Supplementary Figures 1 and 2), as well as the features of the lncRNAs on which they reside in are shown in Supplementary Table 6.

### Results of the replication study

An independent replication of the two SNPs (rs2829145 and rs11171490) potentially implicated in the risk of NAFLD and the disease severity was carried out in a case-control association study that involved a larger dataset of well characterized patients, whose clinical features are shown in Table 2.

**Table 2:** Replication study: Clinical and biochemical characteristics of control subjects and patients with NAFLD

Variables	Control subjects	NAFL	NASH
**Number of subjects**	139	105	146
**Age, years**	45.0 ± 1.4	53.6 ± 1.0	51.8 ± 0.9
**Female %**	62.6	56.4	66.7
**BMI, kg/m**^2^	25.0 ± 0.4	31.6 ± 0.6 *	33.0 ± 0.5 * ^ø^
**Waist circumference, cm**	84.0 ± 1.5	103.0 ± 1.7 *	108.0 ± 1.09 * ^ø^
**Fasting plasma glucose, mg/dL**	81.0 ± 1.0	98.0 ± 4.2 *	130.0 ± 10.4 * ^ø^
**Fasting plasma insulin, μU/ml**	7.0 ± 0.5	12.9 ± 2.0 *	16.4 ± 1.0 * ^ø^
**HOMA-IR index**	1.4.0 ± 0.1	3.1 ± 0.2 *	5.3 ± 0.6 * ^ø^
**SABP, mmHg**	115.0 ± 1.4	125.4 ± 1.7 *	128.0 ± 1.6 *
**DABP, mmHg**	71.5 ± 0.9	77.0 ± 1.3 *	79.0 ± 1.1 *
**Total cholesterol, mg/dL**	209 ± 6	205 ± 5	210 ± 4
**HDL cholesterol, mg/dL**	56 ± 15	52 ± 23	50 ± 14
**LDL cholesterol, mg/dL**	124 ± 38	126 ± 47	125 ± 42
**Triglycerides, mg/dL**	114 ± 11	152 ± 8 *	192 ± 11 *
**AST, U/L**	18.7 ± 1.3	35.0 ± 1.7 *	51.7 * ^ø^
**ALT, U/L**	17 ± 1	57 ± 6 *	72 ± 5 * ^ø^
**GGT, U/L**	24 ± 3	66 ± 6 *	86 ± 8 * ^ø^
**AP, U/L**	140 ± 8	233 ± 10 *	227 ± 10 * ^ø^
**Histological Features**			
**Degree of steatosis, %**	−	48.0 ± 2.5	60.0 ± 1.9 ^ø^
**Lobular inflammation (0–3)**	−	0.6 ± 0.07	1.2 ± 0.06 ^ø^
**Portal inflammation (0–2)**	−	0	1.5 ± 0.06 ^ø^
**Hepatocellular ballooning (0–2)**	−	0	0.8 ± 0.05 ^ø^
**Fibrosis Stage**	−	0	1.5 ± 0.06 ^ø^
**NAS**	−	2.7 ± 0.1	5.9 ± 0.2 ^ø^

Abbreviations: NAFL: nonalcoholic fatty liver, NASH: nonalcoholic steatohepatitis. BMI: body mass index; SABP and DABP: systolic and diastolic arterial blood pressure; HOMA: homeostatic model assessment; ALT and AST: Serum alanine and aspartate aminotransferase; GGT: gamma-glutamyl-transferase; AP: alkaline phosphatase.

Results are expressed as mean ± SD. *P* value stands for statistical significance using Mann-Whitney *U* test, except for female/male proportion that p value stands for statistical significance using *Chi*-*square* test. *Significant difference when compared with controls *p* < 0.05). ^ø^ Significant difference when compared with NAFL (*p* < 0.05).

While the replication study on rs11171490 only demonstrated a significant association between the variant and liver fat content (CC: 50.4 ± 2.0 %, CT: 52.9 ± 3.6 vs. TT: 27.0 ± 12.0, *p* = 0.024 and *p* = 0.033, respectively) and an interaction between sex and the disease severity (*p* = 0.00073), these findings should be interpreted with caution owing to the low frequency of the homozygous T genotype.

On the other hand, the association analysis of rs2829145 confirmed the initial findings. Specifically, in the additive model of inheritance, rs2829145 G/A was significantly associated with NAFLD as disease trait (odds ratio (OR) per A-allele: 1.56 [95% confidence interval (CI) 1.12−2.16], *p* = 0.0081) and NASH (NASH vs. controls: OR per A-allele 2.36 [95% CI 1.54−3.62], *p* = 0.000078 and NASH vs. simple steatosis: OR per A-allele: 1.53 [95% CI 1.04−2.26], *p* = 0.03). The results pertaining to the disease severity remain significant after adjusting for age, sex and body mass index (BMI) (OR per A-allele 1.91 [95% CI 1.05−3.47], *p* = 0.03). The genotype distribution of rs2829145 according to the disease severity was as follows: simple steatosis AA: 8.6%, AG 35.2%, GG 56.2% vs. NASH AA: 13%, AG: 45%, GG: 42%.

In addition, carriers of the A-allele had significantly higher BMI, body fat content, waist/hip ratio and glucose-related phenotypes (including fasting plasma glucose, insulin and HOMA-IR) compared with homozygous GG; complete anthropometric, clinical and biochemical features of the population according to rs2829145 genotypes are shown in Table 3.

**Table 3:** Association analysis with clinical and laboratory features according to rs2829145 genotypes in the recessive model of inheritance

Features	AA + AG	GG	*P* value
**Number of subjects**	179	211	
**Age, years**	49.0 ± 12.5	49.4 ± 12.5	NS
**Female %**	65.4	61.1	NS
**BMI, kg/m**^2^	31.1 ± 6.3	29.9 ± 6.2	0.0007
**Waist circumference, cm**	100.5 ± 19.4	96.2 ± 15.6	0.027
**Waist/hip ratio**	9.93 ± 0.09	0.90 ± 0.09	0.003
**Body fat content, %**	37 ± 8	34 ± 8	0.037
**SABP, mmHg**	122.5 ± 16.4	121.8 ± 15.6	NS
**DABP, mmHg**	76.0 ± 10.7	75.1 ± 11.0	NS
**C reactive protein**	6.2 ± 4.0	6.9 ± 4.7	NS
**Total cholesterol, mg/dL**	208 ± 48	208 ± 41	NS
**HDL cholesterol, mg/dL**	51 ± 16	53 ± 19	NS
**LDL cholesterol, mg/dL**	126 ± 40	124 ± 44	NS
**Triglycerides, mg/dL**	164 ± 98	159 ± 106	NS
**Uric acid, mg/dL**	4.3 ± 2.2	4.4 ± 1.9	NS
**Fasting plasma glucose, mg/dl**	113.7 ± 109.0	97.1 ± 28.0	0.039
**Fasting plasma insulin, μU/ml**	13.0 ± 10.0	10.9 ± 9.0	0.037
**HOMA-IR index**	3.9 ± 6.0	2.8 ± 2.9	0.024
**AST IU/L**	46.7 ± 33.0	34.5 ± 21.3	0.00009
**ALT IU/L**	71 ± 71	46 ± 34	0.00006
**γGT IU/L**	77 ± 80	66 ± 70	NS
**AP IU/L**	238 ± 122	198 ± 99	0.001

After visual inspection of the variables and because of the low frequency of the AA genotype, we followed a recessive model of inheritance; then, comparisons between carriers of the A-allele (AA+AG) versus homozygous GG were performed.

Abbreviations: BMI: body mass index; SABP and DABP: systolic and diastolic arterial blood pressure; HOMA: homeostatic model assessment; ALT and AST: Serum alanine and aspartate aminotransferase; γGT: gamma-glutamyl-transferase; AP: alkaline phosphatase.

Results are expressed as mean ± SD. *P* value stands for statistical significance using Mann-Whitney *U* test, except for female/male proportion that *p* value stands for statistical significance using *Chi*-*square* test.

Exploration of variants in LD with rs2829145 revealed 14 SNPs in strong LD (> 0.8) (Supplementary Table 3), a large proportion of which have significant functionality, as the variants reside in lncRNAs with conserved transcription factor binding sites (TFBS) involved in a myriad of cellular functions, including metabolic processes (Supplementary Table 3).

### Variant in lnc-JAM2-6:4 (rs2829145) and association analysis with circulating miRNAs

Because lncRNAs and miRNAs play an important role in the regulation of cellular processes and available evidence points to a strong interaction between these two types of RNA molecules, we decided to explore potential association between the rs2829145 variant in lnc-JAM2-6:4 and the miRNA expression levels in circulation. Specifically, we tested the hypothesis of an association between rs2829145 genotypes and circulating levels of mir-122, miR-192, miR-375 and the complex miR-19 a/b; these miRNAs were selected because we already found a significant association with NAFLD [24]. In addition, *in silico* analysis of the entire AP000476.1 gene length supports the potential interaction with some of the selected miRNAs (Supplementary Table 6).

**Figure 1:**
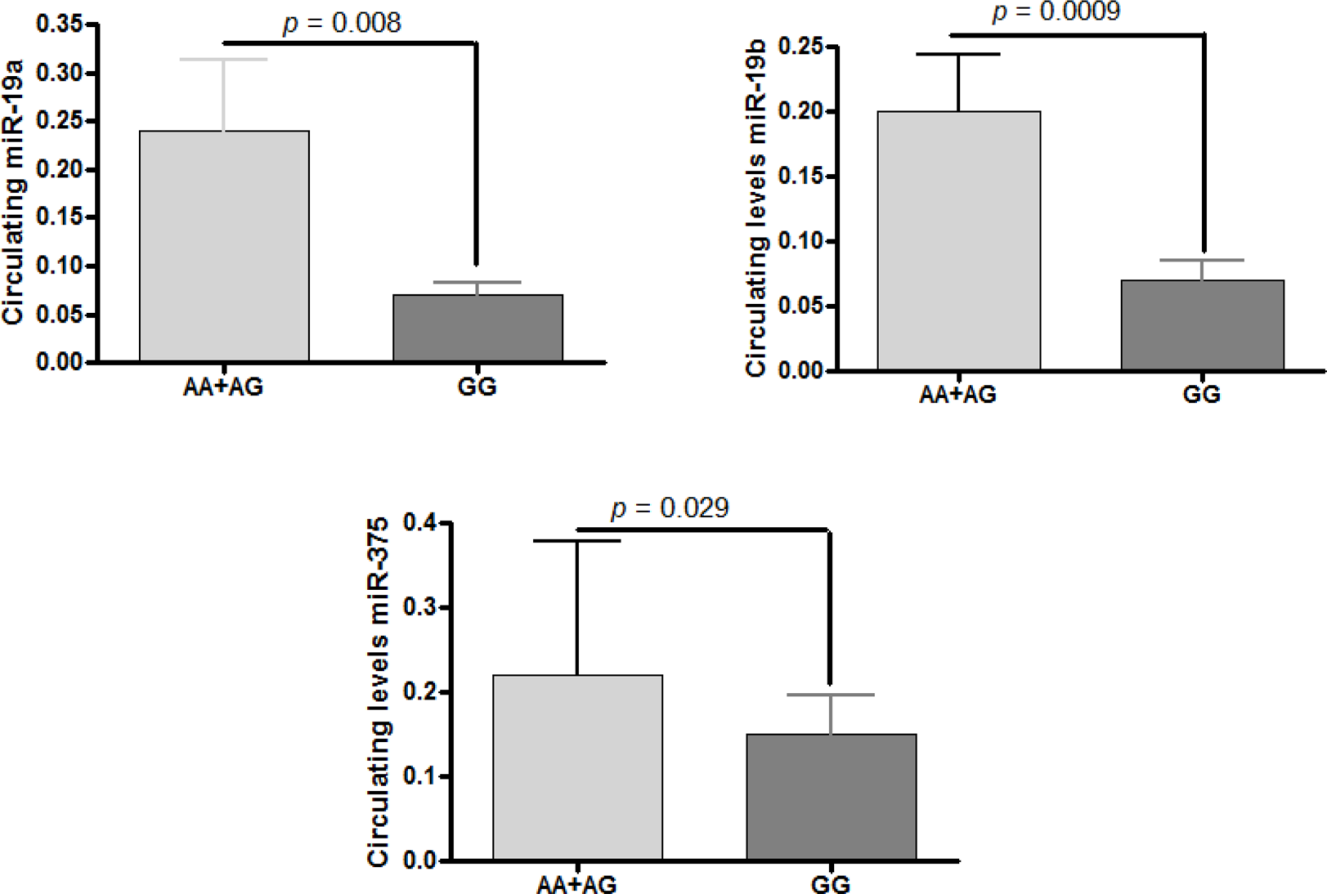
Variant in lnc-JAM2-6:4 (rs2829145) and association analysis with circulating miRNAs The miRNA levels are expressed as the ratio of the estimated amount of the target gene relative to the miR-23a levels [24]. Results are expressed as mean ± SD. The *P* value indicates the statistical significance in the Mann-Whitney *U* test

In the A-allele carriers, we observed increased circulating levels of the complex miR-19a (*p* =0.008) and miR-19b (*p* =0.0009), as well as circulating levels of miR-375 (*p* =0.029) (Figure 1). Though levels of miR-122 and miR-192 in the circulating compartment were higher in the A-allele carriers in comparison with homozygous GG, the differences were not statistically significant.

**Figure 2:**
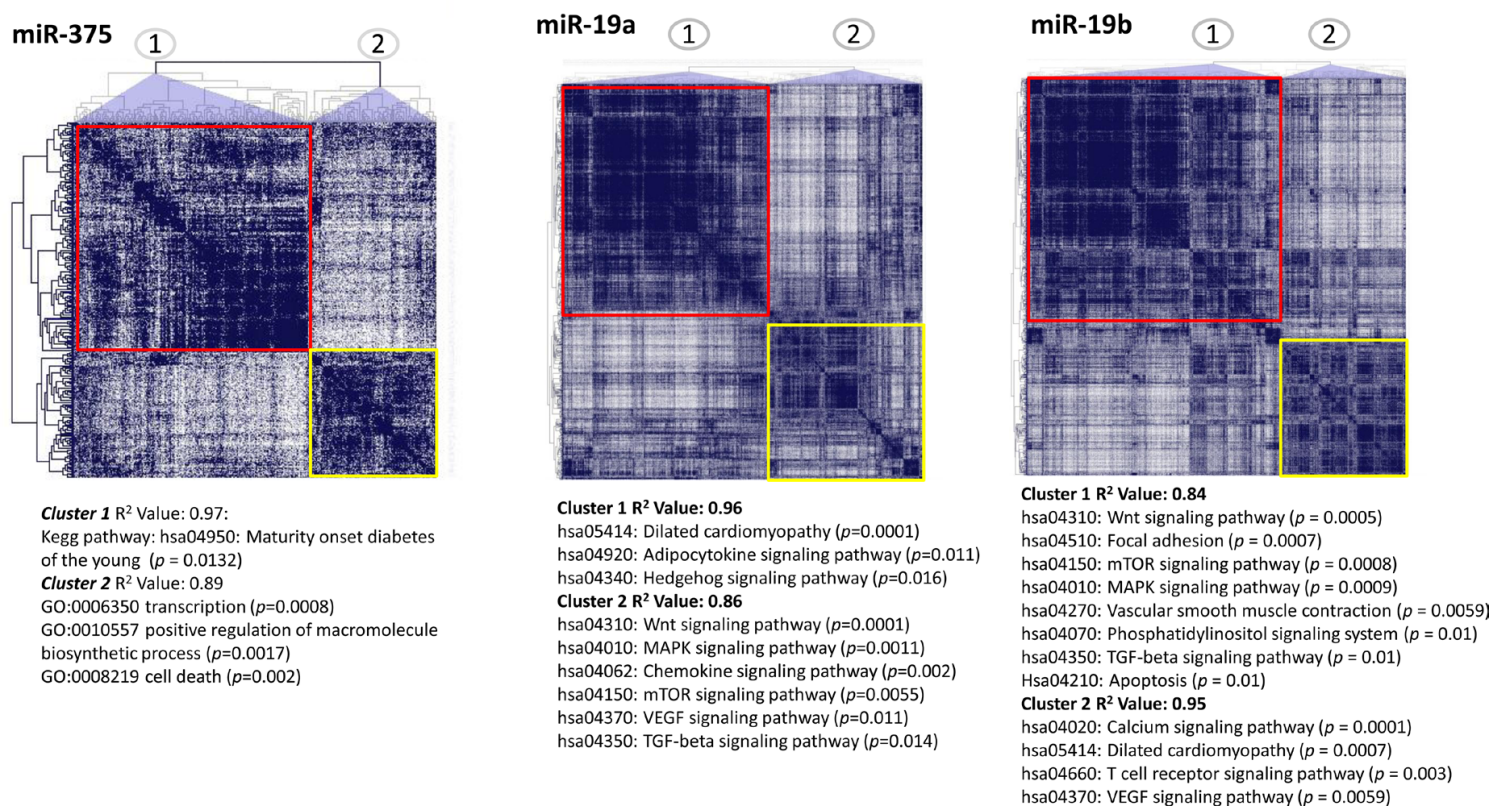
Predicted analysis of target genes, regulatory network and associated functional pathways of miR-375 and the cluster of miR-19a-b The graph represents a heat-map of predicted targets aggregated in co-expression clusters and ranked according to their biological function based on KEGG (Kyoto Encyclopaedia of Genes and Genomes) pathways or Gene Ontology (GO) analysis. KEGG pathways are identified by a combination of a letter code (hsa) and 5 digit number; GO terms are identified by a code of 7 digit numbers. Results are expressed as R-squared [R^2^]; *p* values stand for significance of the enrichment scores expressed as nominal *P*-values < 0.01 and a false discovery rate (FDR) < 0.25. Predicted targets of miR-375 consist of a list of 634 genes, 317 of which falling within the first 50th percentile. Predicted targets of miR-19a and 19b consist of a list of 3577 and 3320 genes, respectively; 1789 and 1660 of which falling within the first 50th percentile, respectively. Predictions were performed by the program CoMeTa (Co-expression Meta-analysis of miRNA Target genes), available at the web site http://cometa.tigem.it.

Further analysis of target prediction and associated pathways showed potential functional explanations of how the rs2829145 variant in *lnc-JAM2-6:4*, as well as the selected miRNAs, could be involved in the pathogenesis of NAFLD. Specifically, miR-375 is predicted to be involved in a KEGG pathway associated with diabetes (Figure 2) and the cluster miR-19 a/b is associated with numerous pathways with a significant role in the biology of NAFLD, including apoptosis, mTOR, MAPK, adipocytokine and hedgehog signaling [2]; a full list of predicted pathways is shown in Figure 2.

## DISCUSSION

In this study, we explored variants located in noncoding genomic regions to understand the potential role of genetic variation in lncRNAs in the pathogenesis of NAFLD. Our findings revealed that rs2829145 was significantly associated with NAFLD as well as the disease severity. The presence of the A-transcript allele was associated with ~ 2-fold and ~1.5-fold increase in the risk of having NASH when compared with control subjects and patients with simple steatosis, respectively. In addition, rs2829145 was associated with obesity and glucose-related traits. Finally, we found a significant association between the rs2829145 variant and the expression levels of target-miRNAs in the circulation, which were previously reported to be strongly associated with NAFLD [24].

The rs2829145 is a common intronic variant (global MAF: A-allele 0.18 with wide variation among ethnicities) that resides in a lncRNA (lnc-JAM2-6); the specific genomic location of this variant is in AP000476.1 gene. While data on functionality of this lincRNA is still lacking, there is compelling information on enriched binding sites in or near the AP000476.1 locus. For example, binding DNA activity with the oncogenes / transcription factors *MAFK* (V-Maf Avian Musculoaponeurotic Fibrosarcoma Oncogene Homolog K), *RAD21* (RAD21 Cohesin Complex Component), *JUND* (Jun D Proto-Oncogene), and *CEBPB* (CCAAT/Enhancer Binding Protein Beta, also known as liver activator protein) has been reported in hepatocellular carcinoma and liver-derived line cells (data extracted from http://www.geneprof.org/). JUND and CEBP are involved in the regulation of the transcriptional activity of hepatic stellate cells, thereby promoting liver fibrosis [25]. Furthermore, there is *in vitro* evidence of methylation and histone modifications in this locus, which are particularly enriched in liver-derived cell lines (Supplementary Table 6). The predicted functional impact of rs2829145 or variants in strong LD points to the involvement of the variant(s) in metabolic processes as well. Collectively, the molecular evidence suggests that rs2829145 variant residing in a lincRNA is a biologically plausible candidate SNP involved in the pathogenesis of NAFLD.

On the other hand, our study suggests the need to evaluate the impact of noncoding regions of the genome on the NAFLD biology. In fact, there is scarce information on the role of variants located in lncRNAs, some of which were sourced from either candidate gene studies [26, 27] or GWAS [10, 28]. For instance, results from a pilot GWAS on NAFLD showed that intergenic or intron variants with predicted functionality in lncRNAs might be associated with the full disease spectrum, including increased lobular inflammation, steatosis score and even liver fibrosis [10]. A complete description of variants in noncoding regions associated with NAFLD and the disease severity in previous studies is shown in Supplementary Table 4.

When interpreting our findings, however, some caveats should be noted because our study is based on analysis of restricted regions of the genome and therefore does not address the unexplored variability within other lncRNA regions. Moreover, the specific role of rs2829145 in the development of the disease requires further generalization in larger datasets. Finally, the assignment of a particular function to the rs2829145 is a challenge due to the presence of a large number of variants in strong LD; hence, we cannot ascertain that rs2829145 is the causal variant. Nevertheless, it is worth nothing that we replicated the initial sequence data in a larger sample of well-characterized patients, which included phenotypic information on liver histology, as well as interactions of the variant not only with clinical outcomes but other regulatory RNA molecules, i.e., miRNAs.

In conclusion, our observations suggest that genetic variation in lncRNAs may contribute to the disease severity, while highlighting the complexity of the genetic component of NAFLD, which involves still unexplored regulatory regions of the genome.

## MATERIALS AND METHODS

### Study design and patient selection

This study was conducted in two phases: (*i*) an initial exploratory study in patients with NAFLD (*n* = 64) and control subjects (*n* = 32) that included a global survey of genetic variation in randomly selected lncRNA-genomic regions located within both experimentally validated and computationally predicted regulatory elements, and (*ii*) independent replication of selected variants in a larger validation set that also involved a case-control design (*n* = 390 participants). Both the exploratory and replication studies involved patients with NAFLD characterized by liver biopsy in addition to other phenotypic traits.

Human serum and DNA samples, well as liver biopsies, were obtained with written informed consent from healthy individuals and those diagnosed with NAFLD following Institutional Review Board-approved protocols. All the investigations performed in this study were conducted in accordance with the guidelines of the 1975 Declaration of Helsinki. Complete details are provided in the Supplemental Content.

### Physical, anthropometric, and biochemical evaluation

Health examinations included anthropometric measurements, a questionnaire on health-related behaviors, and biochemical determinations. Anthropometric measurements and blood samples were obtained from each patient at the time of liver biopsy or DNA sample collection, and prior to any intervention. Detailed information is provided in the Supplemental Content.

### Liver biopsy and histopathological evaluation

Before any intervention, liver biopsy (LB) was performed with ultrasound guidance using a modified 1.4 mm-diameter Menghini needle (Hepafix, Braun, Germany) under local anesthesia, in the outpatient setting. A portion of each liver biopsy specimen was routinely fixed in 40 g/l formaldehyde (pH 7.4) embedded in paraffin before being stained with hematoxylin and eosin, Masson trichrome, and silver impregnation for reticular fibers. All the biopsies were at least 3 cm in length and contained a minimum of eight portal tracts. The liver biopsies were read by an experienced pathologist, who was blinded to all the clinical and laboratory data. The degree of steatosis was assessed according to the system developed by Kleiner et al. [29]; NASH was defined as steatosis, accompanied by mixed inflammatory-cell infiltration, hepatocyte ballooning and necrosis, glycogen nuclei, Mallory's hyaline, and any stage of fibrosis, including absent fibrosis [29, 30]. Full details are provided in the Supplemental Content.

### Exploratory study: Genomic sequencing and bioinformatic analysis of genomic data

NGS was employed for the search of genetic variation in lncRNA-genomic regions, which were examined by semiconductor technology (Ion Torrent PGM™ system IT-PGM) using a 316 chip. All reagents were obtained from the same provider (Life Technologies, Carlsbad, CA, USA). Complete details regarding variant calling, estimation of quality control, data analysis and prediction of variant / mutation effect are provided in the Supplemental Content.

We identified genomic coordinate data (hg19) of human lncRNAs, which was obtained from the UCSC Genome Browser website (https://genome.ucsc.edu/). Human lncRNA-genes coordinates from NONCODEv4 database (www.noncode.org) were intersected with genomic coordinate data (hg19) to obtain annotation details of lncRNA genes, as well as chromosome positions for designing the amplification library. The coordinates of the sequenced regions as well as specific details of predicted functional elements associated with regulation of gene expression are fully disclosed in Supplementary Table 5 and the Supplemental Content. We concomitantly mapped the SNPs that occur in the selected lncRNAs genomic regions; SNPs were identified according to SNP data deposited in dbSNP (build 138) (https://www.ncbi.nlm.nih.gov/SNP/).

### Annotation, prediction and analysis of regulatory elements in the genome

Analysis of regulatory elements in the genome, including genome-wide map of DNase I hypersensitive sites and histone modifications, formaldehyde-assisted isolation of regulatory elements, TFBS, and results of gene expression based on RNA sequencing across a number of cell lines was performed by Encyclopedia of DNA Elements (ENCODE) [31] and Health Roadmap Epigenomics Project [32] (Supplementary Table 1). Detailed information is provided in the Supplemental Content.

### Replication study: genotype and association analysis, power and sample size calculation, and population stratification

The subsequent replication study focused on rs2829145 that is located in a lincRNA (AP000476.1) and rs11171490 that resides in a gene that encodes an antisense transcript (RP11-110A12.2). The genetic analyses were conducted on genomic DNA extracted from white blood cells. Genotyping of rs2829145 *and* rs11171490 was performed using a TaqMan genotyping assay (C__16130085_10 and C_175721423_10; Applied Biosystems, California 92008, USA) according to the manufacturer's instructions.

Using the CaTS power calculator for genetic association studies [33] and assuming a prevalence of NAFLD of 0.30, a MAF of ~0.2 and a relative risk of ~1.5, our sample had 91% power for the additive genetic model of rs2829145. Population stratification assessment was conducted as previously described [34].

### Circulating miRNA expression

We explored circulating expression levels of targeted miRNAs in a sample of 86 subjects, including 68 patients with NAFLD and 18 healthy volunteers. Details on miRNA isolation and quantification by real-time quantitative reverse-transcription PCR (RT-PCR) assay, as well as primer sequences, are published elsewhere [24]. The results of serum miRNA expression were normalized to the most stable reference miRNA [24].

### Statistical analysis

Quantitative data were expressed as mean ± SD unless otherwise indicated. Because significant differences in variance were observed between the groups in most of the variables and the distribution was significantly skewed in most cases, we chose to be conservative and assessed the differences between the group means by using nonparametric Mann-Whitney *U* or Kruskal-Wallis tests. We used the *a priori* additive genetic model of inheritance unless indicated otherwise.

The Cochran–Armitage test for trend or logistic regression was employed in the categorical data analysis to assess the presence of association between the variant and dichotomized disease severity, while we performed a regression analysis for an ordinal multinomial distribution (Probit as the Link function) with disease severity as the dependent variable (response with more than two categories). The controls, NAFL and NASH subjects were coded as 0, 1, and 2, respectively, and the analyses adjusted for cofounders such as age, sex and BMI when indicated. The CSS/Statistica program package version 6.0 (StatSoft, Tulsa, OK, USA) was employed in the aforementioned analyses.

## SUPPLEMENTARY MATERIALS FIGURES AND TABLES











## Abbreviations

BMI: body mass index
CI: confidence interval
GWAS: genome-wide association study
LncRNAs: long noncoding RNAs
LincRNA: long intergenic noncoding RNA
MAF: minor allele-frequency
NAFLD: nonalcoholic fatty liver disease
NAFL: nonalcoholic fatty liver
NASH: nonalcoholic steatohepatitis
NGS: next generation sequencing
OR: odds ratio
SNP: single nucleotide polymorphism
TFBS: transcription factor binding sites
