# Supplementary material for: Genetic variation in long noncoding RNAs and the risk of nonalcoholic fatty liver disease
Source: Oncotarget. 2017 Feb 11;8(14):22917–26. doi: 10.18632/oncotarget.15286 (PMC5410273; doi:10.18632/oncotarget.15286)
Supplement: Supplementary file 1 [file oncotarget-08-22917-s001.pdf]

Genetic variation in long noncoding RNAs and the risk of nonalcoholic fatty liver disease

Supplementary Materials

MATERIALS AND METHODS

Study design and patient selection

Patients were included in the study if there was histopathological evidence of NAFLD, either NAFL or NASH, based on a liver biopsy conducted within the study period. Exclusion criteria were secondary causes of steatosis, including alcohol abuse (≥ 30 g alcohol daily for men and ≥ 20 g for women), total parenteral nutrition, hepatitis B and hepatitis C virus infection, and the use of drugs known to precipitate steatosis. By using standard clinical and laboratory evaluation, as well as liver biopsy features when applicable, autoimmune liver disease, metabolic liver disease, Wilson's disease, and α-1-antitrypsin deficiency were likewise ruled out in all patients.

Healthy subjects were selected for inclusion into the control group if their age and sex matched those of the NAFLD patients and in whom, in addition to the standard health assessment described below, a careful *ultrasonographic* (US) examination of the *liver* was performed to exclude fatty liver infiltration. In addition, controls were included at any study phase if they did not exhibit features of MetS and did not have fatty liver at liver US.

The case participants and the controls were selected during the same study period from the same population of patients attending the Liver Unit, and all shared the same demographic characteristics.

Liver biopsy and histopathological evaluation

The degree of steatosis was assessed according to the system developed by Kleiner et al.[29] based on the percentage of hepatocytes containing macrovesicular fat droplets: grade 0 = < 5%; grade 1 = 5-33%; grade 2 = 34% to 66% and grade 3 = > 66%. NASH was defined as steatosis, accompanied by mixed inflammatory-cell infiltration, hepatocyte ballooning and necrosis, glycogen nuclei, Mallory's hyaline, and any stage of fibrosis, including absent fibrosis [30]. Intra-acinar (lobular) inflammation was defined according to Brunt [30] as presence of cellular components of inflammation (polymorphonuclear leukocytes, lymphocytes and other mononuclear cells, eosinophils and microgranulomas) located in sinusoidal spaces, surrounding Mallory's hyaline or in hepatocellular necrosis. It was graded 0–3 and was defined as 0 (absent) = no foci; 1 = < 2 foci per 200 × field; 2 = 2 to 4 foci per 200 × field; and 3 = >4 foci per 200 × field. Ballooning was scored as: 0 = none; 1 = rare or few; and 2 = many. The severity of fibrosis was expressed on a 4-point scale, as follows: 0 = none; 1 =perivenular and/or perisinusoidal fibrosis in zone 3; 2 = combined pericellular portal fibrosis; 3 = septal/ bridging fibrosis; and 4 = cirrhosis [29, 30].

Next generation sequencing (NGS)

DNA was isolated from whole blood, as previously described [26,27,34] and was quantified by a Qubit DNA high-sensitivity assay kit (Life Technologies, Carlsbad, CA, USA).

Library preparation for each sample was performed using the IT AmpliSeq 2.0 Beta kit following the manufacturer's instructions (Life Technologies, Carlsbad, CA, USA). Briefly, 10 ng of DNA was used as a template to generate the amplicon library for sequencing variation in the selected regions by the Ampliseq software (Life Technologies, Carlsbad, CA, USA). Genomic regions of interest were PCR amplified prior to sequencing, and the sequencing adaptors with short stretches of index sequences (96 barcodes) that enabled sample identification were ligated to the amplicons using the IT Xpress barcode adaptor kit. The prepared library was quantified using the Ion library TaqMan Quantitation Kit. Sequencing template preparation (emulsion PCR and beads-enrichment) from sequencing libraries was carried out using an Ion OneTouch Template Kit and Ion OneTouch system (Ion OneTouch Instrument and Ion OneTouch

ES, Life Technologies, Carlsbad, CA, USA) according to the manufacturer's protocol. Prepared templates were sequenced using Ion Sequencing Kit v2. Ion Torrent Suite software 4.2.1 (Life Technologies, Carlsbad, CA, USA) was used for converting raw signals into base calls and extracting FASTQ files of sequencing reads. Number of nucleotide flows during sequencing was set to 500 cycles.

Variant calling, estimation of quality control, data analysis and prediction of variant/ mutation effect

The data obtained from the Ion Torrent PGM were processed using the Ion Torrent Suite Software v 4.2.1 (Life Technologies, Carlsbad, CA, USA). Variants were annotated with dbSNP (http://www.ncbi.nlm.nih.gov/SNP/) IDs using SnpSift. *In silico* analysis, aimed at predicting gene and transcript functional consequences was performed by the bioinformatic tool variant Effect Predictor (VEP) (http://useast.ensembl.org/Homo_sapiens/Tools/VEP), employing ENSEMBL transcripts and SnpEff platform (http://snpeff.sourceforge.net/) using University of California, Santa Cruz (UCSC) transcripts. CellBase available at http://docs.bioinfo.cipf.es/projects/cellbase was used to annotate variants with the phenotype information from HGMD, ClinVar, UNIPROT and COSMIC. Annotation in http://www.ncbi.nlm.nih.gov/SNP was also used to determine whether variants were novel or already associated with a phenotype.

Read alignment to the hg19 Human genome reference sequence was performed using torrent mapping alignment program (TMAP) included in PGM. The Ion Torrent variant caller plug-in was used to perform the variant calling with the germline low stringency settings. In order to filter erroneous base callings, control quality filtering steps were performed using proprietary Perl scripts. At the variant coverage depth > 20, we inspected the presence of strand bias by checking the number of bases covered in both strands for the variant, the reference >= 4 reads in both strands, and the quality for variant calling > 17. The retained variants were visually examined using Integrative Genomics Viewer (IGV) software (http://www.broadinstitute.org/igv/) to check for any inconsistency in the base calls.

Annotation, prediction and analysis of regulatory elements in the genome

Specific information of lncRNAs, including annotation and position in the genome, predicted functionality, expression profiling, methylation sites and chromatin modifications, and protein/s and miRNAs (micro-RNAs) interactions was additionally performed by the following datasets available online: http://www.lncipedia.org/db (last version May 2016), http://www.noncode.org/, Human body map 2.0 (http://www.ensembl.info/blog/2011/05/24/human-bodymap-2-0-data-from-illumina/, http://lncrnator.ewha.ac.kr/, http://starbase.sysu.edu.cn/, MirTarget2, http://genome.igib.res.in/lncRNome/. ALE-HSA21, available at http://bioinfo.na.iac.cnr.it/, was used for computational analysis of interaction between noncoding transcripts and miRNAs specifically from chromosome 21 (further details are provided thereafter).

RegulomeDB, a database that annotates SNPs with known and predicted regulatory elements in the intergenic regions of the H. sapiens genome, was used for the prediction of variants effects' (http://www.regulomedb.org/snp/). HaploReg tool, available at http://compbio.mit.edu/HaploReg, was used for exploring regulatory elements in the selected SNP/s, including information of variants in linkage disequilibrium (LD) from the 1000 Genomes Project, linked SNPs and small indels along with chromatin state and protein binding annotation from the Roadmap Epigenomics and ENCODE projects, and the effect of SNPs on regulatory motifs.

Prediction of functional elements potentially associated with regulation of gene expression in the sequenced regions

We focused on the following data: (1) ENCODE Project transcription factors binding sites (TFBS) for HepG2 cell line and (2) Roadmap Epigenomics Project 15-state chromatin segmentations for various cell lines (HepG2, adult liver, adipose nuclei, heart left ventricle, heart right ventricle and heart right atrium). Each region of interest was bioinformatically assessed using AnnotationHub, an R package available through Bioconductor as explained elsewhere [18].

The ENCODE (En**c**yclopedia Of DNA Elements) project (https://www.genome.gov/encode/) was used to predict TFBS; the cell type selected was HepG2 (which is a cell line derived from a male patient with liver carcinoma). The rationale of this selection is: 1- our work was focused on the liver, 2-this is a model system for metabolism disorders, 3-the cell line represents the endoderm lineage, 4-ENCODE only contains information on designated cell types.

TFBS data is based upon ChIP-seq experiments and consists of peak calls (regions of enrichment) based on an uniform processing pipeline developed for the ENCODE Integrative Analysis effort. The score values were computed at UCSC based on signal values assigned by the ENCODE uniform analysis pipeline. The input signal values were multiplied by a normalization factor calculated as the ratio of the maximum score value (1000) to the signal value at 1 standard deviation from the mean, with values exceeding 1000 capped at 1000. This has the effect of distributing scores up to mean + 1std across the score range, but assigning all above to the maximum score.

The NIH Roadmap Epigenomics Mapping Consortium (http://www.roadmapepigenomics.org/) was used to predict information on chromatin accessibility

in the sequenced regions chromatin segmentations. Chromating state learning data is based upon different chromatin marks (H3K4me3, H3K4me1, H3K36me3, H3K27me3, H3K9me3) in their spatial context (chromatin states) across the epigenome that were analyzed with ChroHMM v1.10 by the Roadmap Epigenomics Project. The trained model was then used to compute the posterior probability of each state for each genomic bin in each cell type. Our regions of interest were labeled using the state with the maximum posterior probability.

Prediction of miRNAs target genes and pathway analysis

The Co-expression Meta-analysis of miRNA Targets (CoMeTa) available at http://cometa.tigem.it/index.php was used to predict target genes of miRNAs as well as pathway analysis. This platform is based on the assumption that the targets of a given miRNA are likely to be co-expressed and therefore to belong to the same miRNA gene network. The CoMeTa tool aims at the inference of miRNA targets and miRNA-regulated gene networks by integrating expression data from hundreds of cellular and tissue conditions.

CoMeTa integrates expression data from hundreds of cellular systems and multiple tissues along with the analysis of 675 human miRNAs.

Supplementary Table 1: Complete details of lncRNAs genomic regions sequenced in the exploratory study by utilizing next generation sequencing technology. See Supplementary_Table_1

Supplementary Table 2: Complete details of SNPs sequenced in the exploratory study utilizing next generation sequencing technology. See Supplementary_Table_2

Supplementary Table 3: Exploration of variants in linkage disequilibrium (LD) with rs2829145 and predicted regulatory functionality

chromosome	Genomic position (hg38)	LD (r^2)	LD (D')	Variant ID	Reference allele	Alternative allele	Motifs changed	GENCODE genes
21	24495924	**1**	**1**	rs2829145	G	A	GATA	AP000476.1
21	24497997	**1**	**1**	rs2186436	G	C	–	AP000476.1
21	24493912	**1**	**1**	rs2829135	G	A	4 altered motifs	AP000476.1
21	24498376	**1**	**1**	rs2186438	C	T	8 altered motifs	AP000476.1
21	24499310	**1**	**1**	rs75275867	C	G	Pax-4, STAT, Smad	AP000476.1
21	24499837	**1**	**1**	rs36003834	CA	C	Glis2, Nanog, VDR	AP000476.1
21	24504470	**0.99**	**1**	rs28565163	T	C,G	–	AP000476.1
21	24505956	**0.97**	**1**	rs149955684	TTCA	T	Brachyury, Myc	AP000476.1
21	24507259	**1**	**1**	rs150998654	G	A		AP000476.1
21	24508080	**0.89**	**1**	rs12053678	T	C	6 altered motifs	AP000476.1
21	24509482	**0.91**	**1**	rs12626970	C	T	19 altered motifs	AP000476.1
21	24518517	**0.98**	**0.99**	rs73141668	T	C	5 altered motifs	AP000476.1
21	24519345	**0.98**	**0.99**	rs73141670	A	G	BCL,HNF1,PU.1	AP000476.1
21	24523015	**0.97**	**0.99**	rs2829151	C	T	5 altered motifs	AP000476.1

Prediction was performed by The HaploReg database available at http://compbio.mit.edu/HaploReg. In red font is highlighted the variant of interest. Motifs changes stands for potential identification of active regulatory elements associated with the variant, including conserved TFBS (transcription factors binding sites).

Supplementary Table 4: The role of variants in lncRNAs regions in the pathogenesis of NAFLD: Single nucleotide polymorphisms (SNPs) from GWAS for NAFLD

SNP ID/alleles	Gene	Predicted functionality	Transcrip (strand) / allele	MAF	NAFLD trait	Reference
rs2645424<br>A/C/G<br>Ancestral: G	FDFT1	Non coding transcript variant<br><br>NMD transcript variant	ENST00000446331 (+)/ C<br>ENST00000446331 (+)/G<br><br>ENST00000525283 (+)/ C<br>ENST00000525283 (+)/G	0.48	NAS Score	10
rs343064<br>C/T<br>Ancestral: C	-	lincRNA	ENST00000441150 (-)/ TA	0.35	fibrosis	10
rs1227756<br>G/A<br>Ancestral: A	COL13A1	NMD transcript variant	ENST00000479733 (+)/A	0.39	Lob. inflamm	10
rs887304<br>T/C<br>Ancestral: C	EFCAB4B	NMD transcript variant	ENST00000333750<br>(-)/G	0.14	Lob. Inflamm	10
rs12137855<br>C/T<br>Ancestral: A	LYPLAL1	lincRNA	ENST00000612055 (+)/T	0.16	Liver fat	28
rs4240624<br>G/A<br>Ancestral: A	PPP1R3B	lincRNA	ENST00000518619 (+)/A<br><br>ENST00000520255 (+)/A<br>ENST00000520390 (+)/A	0.11	Liver fat	28
rs2954021<br>A/G<br>Ancestral: A	TRIBI	lincRNA	ENST00000522815 (+)/G	0.45	Liver fat	28
rs2126259<br>T/C<br>Ancestral: C	PPP1R3B	lincRNA	ENST00000518619 (+)/C<br>ENST00000520255 (+)/C<br>ENST00000520390 (+)/C	0.12	Liver fat and inflammation	11

MAF: minor allele frequency (http://www.ensembl.org/).
NMD: nonsense mediated decay.
Lob. Inflamm: lobular inflammation.

Supplementary Table 5: The coordinates (chromosome location based on genomic coordinate data GRCh37/hg19) of the sequenced regions as well as details on predicted functional elements associated with regulation of gene expression. See Supplementary_Table_5

Supplementary Table 6: Features of SNPs in lncRNAs-regions associated with NAFLD in the exploratory study and further followed-up in the replication stage. See Supplementary_Table_6

Gene: AP000476.1 (ENSG00000237484)

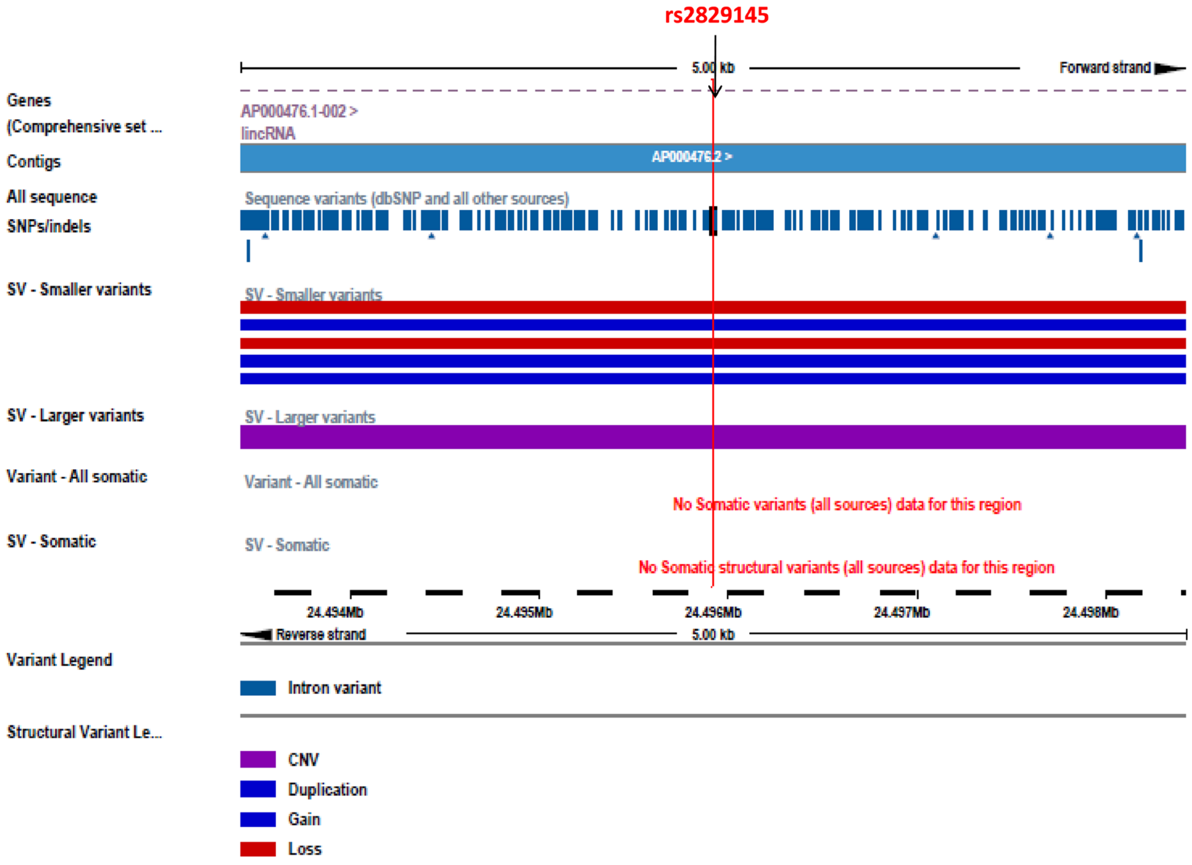

Supplementary Figure 1: AP000476.1: Gene location and genomic details of rs2829145. The picture shows the genomic assembly as a blue bar (composed of individual contigs). The rs2829145 (outlined by a red vertical line) is shown in a 5 kb region along with surrounding variations, which are staggered in multiple rows for ease of viewing. A legend at the bottom indicates which colors are used for the different variation consequence types. In addition, transcripts and regulatory features annotated in this region are also displayed.

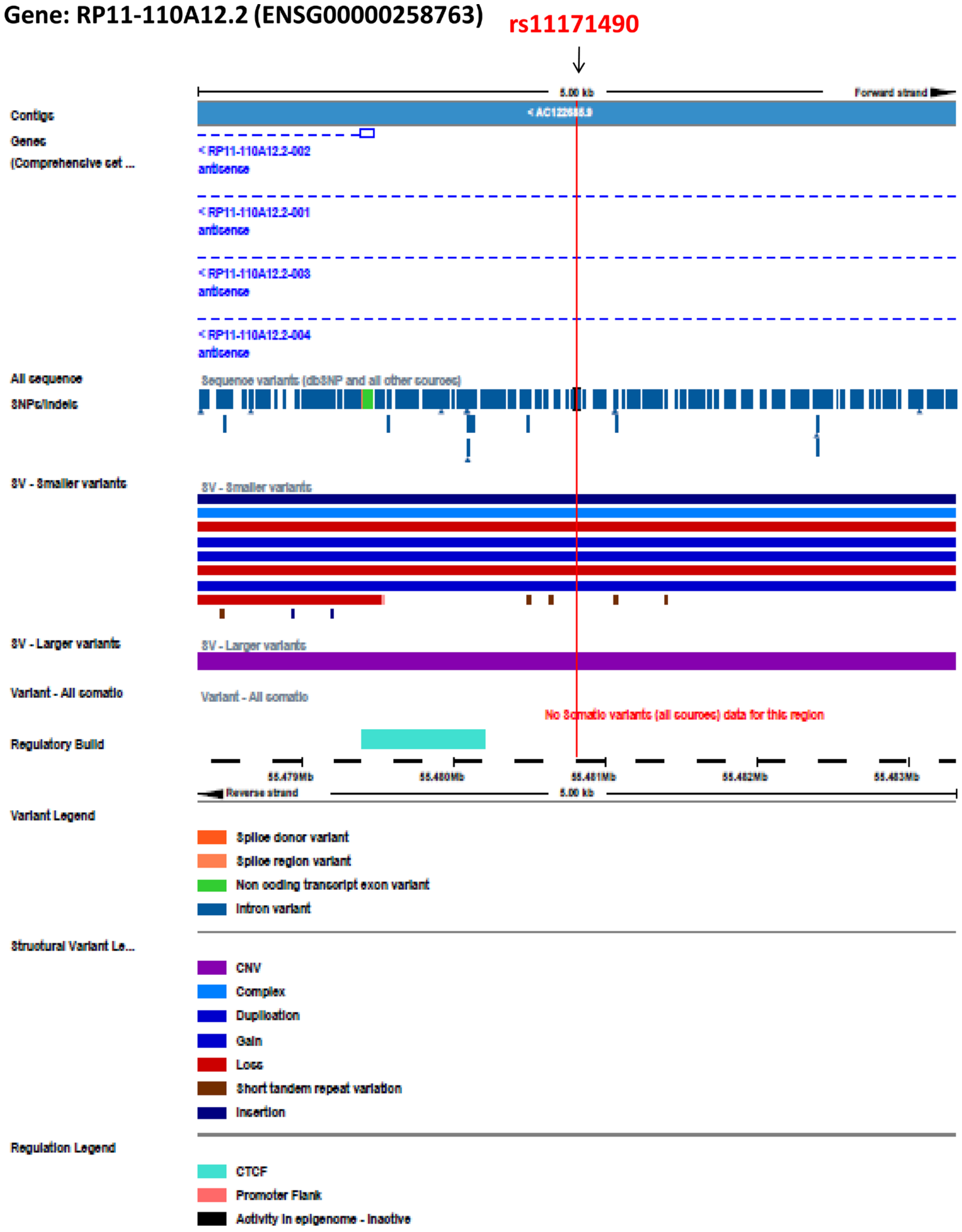

Supplementary Figure 2: RP11-110A12.2: Gene location and genomic details of rs11171490. The picture shows the genomic assembly as a blue bar (composed of individual contigs). The rs11171490 (outlined by a red vertical line) is shown in a 5 kb region along with surrounding variations, which are staggered in multiple rows for ease of viewing. A legend at the bottom indicates which colors are used for the different variation consequence types. In addition, transcripts and regulatory features annotated in this region are also displayed.
