# Supplementary material for: Genetic variation in long noncoding RNAs and the risk of nonalcoholic fatty liver disease
Source: Oncotarget. 2017 Feb 11;8(14):22917–26. doi: 10.18632/oncotarget.15286 (PMC5410273; doi:10.18632/oncotarget.15286)
Supplement: Supplementary file 2 [file oncotarget-08-22917-s002.docx]

**Supplementary Table 1**

**Complete details of lncRNAs annotation names/ ID number sequenced in the exploratory study by next generation sequencing technology**

| **lncRNA ID**  **(noncode database#)** |
| --- |
| NONHSAG002533  NONHSAG002534  NONHSAT005435  NONHSAT005437  NONHSAT005439  NONHSAT005441  NONHSAT005442  NONHSAT005444  NONHSAT005445 |
| NONHSAG034746  NONHSAG036890  NONHSAG036892  NONHSAG036975  NONHSAT088970  NONHSAT088972  NONHSAT093781  NONHSAT093783  NONHSAT093962 |
| NONHSAG041903  NONHSAT104491  NONHSAT104492  NONHSAT104493  NONHSAT104494  NONHSAT104495  NONHSAT104496  NONHSAT104497  NONHSAT104498  NONHSAT104499  NONHSAT104500  NONHSAT104501 |
| NONHSAG047030  NONHSAG047253  NONHSAG047359  NONHSAT119356  NONHSAT119857  NONHSAT147544 |
| NONHSAG051258  NONHSAG051259  NONHSAT129048  NONHSAT129049  NONHSAT129051  NONHSAT129052  NONHSAT129053  NONHSAT129054  NONHSAT129055  NONHSAT129056  NONHSAT129058  NONHSAT129059  NONHSAT129060  NONHSAT129061  NONHSAT129062  NONHSAT129063  NONHSAT129074  NONHSAT129075  NONHSAT129078  NONHSAT129082  NONHSAT129083  NONHSAT129084  NONHSAT147662 |
| NONHSAG051691  NONHSAT130069 |
| NONHSAG007404  NONHSAG008482  NONHSAG008617  NONHSAG009343  NONHSAT017449  NONHSAT021686  NONHSAT022004  NONHSAT023587  NONHSAT023588  NONHSAT023590  NONHSAT023591 |
| NONHSAG010338  NONHSAG010534  NONHSAG011311  NONHSAG011590  NONHSAG011763  NONHSAG011935  NONHSAT026154  NONHSAT026155  NONHSAT026156  NONHSAT026157  NONHSAT026158  NONHSAT026996  NONHSAT028661  NONHSAT028662  NONHSAT028663  NONHSAT029258  NONHSAT029619  NONHSAT029620  NONHSAT029966 |
| NONHSAG013937  NONHSAT034645 |
| NONHSAG014611  NONHSAG015878  NONHSAG015883  NONHSAG015884  NONHSAG016005  NONHSAT036216  NONHSAT039634  NONHSAT039643  NONHSAT039650  NONHSAT039651  NONHSAT040146  NONHSAT040147  NONHSAT040148 |
| NONHSAG016942  NONHSAG017074  NONHSAG017780  NONHSAT043957  NONHSAT044215  NONHSAT044217  NONHSAT048141 |
| NONHSAG018667  NONHSAT140673 |
| NONHSAG020469  NONHSAG020480  NONHSAG021099  NONHSAG022012  NONHSAG022898  NONHSAT054186  NONHSAT054187  NONHSAT056242  NONHSAT056244  NONHSAT056245  NONHSAT144847  NONHSAT144881  NONHSAT146451 |
| NONHSAG026451  NONHSAG026719  NONHSAG026724  NONHSAG026725  NONHSAG026726  NONHSAG026730  NONHSAT067657  NONHSAT068368  NONHSAT068369  NONHSAT068370  NONHSAT068371  NONHSAT068372  NONHSAT068373  NONHSAT068384  NONHSAT068385  NONHSAT068386  NONHSAT068387  NONHSAT068388  NONHSAT068436 |
| NONHSAG032137  NONHSAG032138  NONHSAG032205  NONHSAT080538  NONHSAT080539  NONHSAT080697 |
| NONHSAG032538  NONHSAT081453 |
| NONHSAG034112  NONHSAT087160 |
| NONHSAG054788  NONHSAG054830  NONHSAT137581  NONHSAT137588  NONHSAT137589  NONHSAT137593  NONHSAT137598  NONHSAT137601  NONHSAT137603  NONHSAT137604  NONHSAT137606  NONHSAT137607  NONHSAT137681 |

#: NONCODE: http://www.noncode.org/
