# Supplementary material for: Genetic variation in long noncoding RNAs and the risk of nonalcoholic fatty liver disease
Source: Oncotarget. 2017 Feb 11;8(14):22917–26. doi: 10.18632/oncotarget.15286 (PMC5410273; doi:10.18632/oncotarget.15286)
Supplement: Supplementary file 3 [file oncotarget-08-22917-s003.docx]

**Supplementary Table 2**

**Complete details of single nucleotide polymorphisms (SNPs) sequenced in the exploratory study by next generation sequencing technology**

| **Chromosome** | **Genomic coordinates** | **SNP ID dbSNP #** | **Reference allele** | **Alternate allele** | **MAF *** |
| --- | --- | --- | --- | --- | --- |
| chr17 | 1900039 | . | A | G |  |
| chr12 | 6943251 | . | T | C |  |
| chr16 | 14310702 | . | C | T |  |
| chr17 | 44012159 | . | G | A |  |
| chr17 | 44012260 | . | T | C | 0.1056 |
| chr22 | 44887362 | . | C | T |  |
| chr15 | 53452550 | . | G | A |  |
| chr19 | 53755307 | . | A | G |  |
| chr19 | 53755312 | . | G | A |  |
| chr19 | 53755380 | . | C | G |  |
| chr19 | 58953351 | . | C | A |  |
| chrX | 73423865 | . | G | A |  |
| chrX | 76142290 | . | C | A |  |
| chr11 | 90242025 | . | C | T |  |
| chr13 | 90801288 | . | C | T |  |
| chr14 | 100601803 | . | C | G |  |
| chr3 | 191030508 | . | C | A |  |
| chr12 | 12960007 | rs10542779 | GAGCT | G | 0.0418 |
| chr9 | 4840380 | rs10974808 | A | G | 0.0579 |
| chr12 | 6943243 | rs11064425 | T | C | 0.0147 |
| chr12 | 55874598 | rs11171490 | C | T | 0.0840 |
| chr13 | 90801538 | rs11354678 | TC | T | 0.0147 |
| chr11 | 61316529 | rs114204482 | C | T | 0.0138 |
| chr14 | 100584805 | rs117696874 | C | A | 0.0060 |
| chr14 | 100588485 | rs11849414 | G | A | 0.0657 |
| chr1 | 117015884 | rs12406799 | G | C | 0.0115 |
| chr15 | 60903316 | rs12905398 | G | C | 0.0028 |
| chr19 | 58956326 | rs12980907 | C | T | 0.1483 |
| chr20 | 56826020 | rs13040116 | A | G | 0.0399 |
| chr12 | 78337175 | rs1382638 | G | A | 0.4936 |
| chr8 | 128877343 | rs140433039 | G | T | 0.0023 |
| chr5 | 148790471 | rs140976932 | C | G | 0.0110 |
| chr14 | 103653569 | rs142226249 | CTT | C | 0.0165 |
| chr19 | 58953224 | rs142797717 | G | A | 0.0060 |
| chr19 | 58861808 | rs145144275 | A | G |  |
| chr19 | 58920551 | rs147393087 | C | G | 0.0051 |
| chr7 | 18133392 | rs150090468 | G | A | 0.0096 |
| chr20 | 56826668 | rs1537479 | C | T | 0.4706 |
| chrX | 76142263 | rs16992337 | A | G | 0.0750 |
| chr21 | 25868188 | rs16999980 | A | C | 0.0519 |
| chr15 | 60903348 | rs17237304 | C | T | 0.0693 |
| chr19 | 53755325 | rs184075423 | A | G | 0.0009 |
| chr15 | 87712291 | rs185469904 | C | T | 0.0018 |
| chr8 | 129090402 | rs1875025 | T | C | 0.3398 |
| chr16 | 14310621 | rs192269577 | A | G | 0.0009 |
| chr17 | 1563897 | rs202160704 | A | G | 0.0005 |
| chr11 | 1974542 | rs217206 | G | A | 0.4844 |
| chr12 | 67953251 | rs2178427 | C | T | 0.1736 |
| chr19 | 58906163 | rs2241787 | T | C | 0.4679 |
| chr19 | 58911768 | rs2278498 | A | G | 0.4747 |
| chr20 | 60572663 | rs2296081 | C | A | 0.3792 |
| chr21 | 25868238 | rs2829145 | G | A | 0.2020 |
| chr12 | 94226328 | rs34711247 | CTT | C | 0.1299 |
| chr14 | 103653579 | rs3742451 | G | T | 0.0243 |
| chr9 | 4840388 | rs376777472 | TTGAAG | T | 0.0257 |
| chr9 | 4840354 | rs377208552 | A | G |  |
| chr20 | 60572568 | rs41284980 | G | A | 0.0312 |
| chr14 | 100583491 | rs4290419 | G | C | 0.3972 |
| chr17 | 76713750 | rs4796807 | G | A | 0.1148 |
| chr17 | 19188381 | rs4924971 | T | C | 0.0895 |
| chr11 | 90241946 | rs558396 | C | T | 0.4169 |
| chr14 | 100585755 | rs57612702 | C | T | 0.0914 |
| chr14 | 30553732 | rs57729441 | T | C | 0.2397 |
| chr14 | 30553607 | rs58947498 | A | C | 0.2397 |
| chrX | 76142231 | rs5937645 | T | A | 0.1167 |
| chr17 | 44012096 | rs62061716 | A | G | 0.1162 |
| chr17 | 44012248 | rs62061717 | T | A | 0.1157 |
| chr17 | 1563885 | rs62088058 | C | T | 0.0174 |
| chr19 | 58953319 | rs6510152 | C | T | 0.4330 |
| chr17 | 44012257 | rs66755419 | CG | C | 0.1056 |
| chr7 | 30296022 | rs6949732 | C | T | 0.1286 |
| chr11 | 64415409 | rs71579862 | A | T | 0.0119 |
| chr14 | 100583349 | rs7158754 | A | G | 0.1869 |
| chr14 | 100583330 | rs7159195 | G | A | 0.1869 |
| chr19 | 53755311 | rs7259373 | C | T | 0.0101 |
| chr14 | 100601444 | rs72711957 | T | G | 0.1364 |
| chr7 | 36925535 | rs73119923 | T | C | 0.1593 |
| chr12 | 67953226 | rs73130322 | T | C | 0.0133 |
| chr12 | 67953178 | rs73330271 | A | T | 0.0592 |
| chr14 | 100584776 | rs73349550 | T | C | 0.0220 |
| chr15 | 53452349 | rs73405909 | G | C | 0.0762 |
| chr17 | 1563889 | rs73976249 | A | C | 0.0675 |
| chr12 | 12960069 | rs74063805 | C | A | 0.0119 |
| chr14 | 100595880 | rs74085121 | G | A | 0.0643 |
| chr3 | 185441877 | rs74452831 | A | G | 0.0510 |
| chr3 | 35761038 | rs74936769 | C | T | 0.0087 |
| chr3 | 191030544 | rs76956371 | T | C | 0.0248 |
| chr14 | 100592351 | rs77811913 | G | A | 0.0358 |
| chr17 | 1900020 | rs78621507 | C | T | 0.0197 |
| chr7 | 30296049 | rs78773371 | A | G | 0.0381 |
| chr11 | 90241991 | rs78840388 | G | A | 0.0069 |
| chr19 | 58956217 | rs893189 | C | G | 0.1478 |
| chr3 | 35761070 | rs9824070 | C | G | 0.1244 |

*MAF: minor allele frequency; values stand for intra-study MAF.

### #: dbSNP: the NCBI database of genetic variation available at: <https://www.ncbi.nlm.nih.gov/SNP/>
