# Supplementary material for: Genetic variation in long noncoding RNAs and the risk of nonalcoholic fatty liver disease
Source: Oncotarget. 2017 Feb 11;8(14):22917–26. doi: 10.18632/oncotarget.15286 (PMC5410273; doi:10.18632/oncotarget.15286)
Supplement: Supplementary file 4 [file oncotarget-08-22917-s004.docx]

**Supplementary Table S5**

**The coordinates (chromosome location based on genomic coordinate data GRCh37/hg19) of the sequenced regions as well as details on predicted functional elements associated with regulation of gene expression**

| **Regions of Interest** | | | **ENCODE (TFBS HepG2)** | | **Roadmap Epigenomics (Chromatin Segmentations)** | | | | | |
| --- | --- | --- | --- | --- | --- | --- | --- | --- | --- | --- |
| **Chr.** | **Start** | **End** | **TF score** | **TF name** | **HepG2** | **Adult Liver** | **Adipose Nuclei** | **Heart Left Ventricle** | **Heart Right Ventricle** | **Heart Right Atrium** |
| chr1 | 1092346 | 1092441 |  |  | Enhancers | Weak Repressed PolyComb | Quiescent/Low | Quiescent/Low \| Enhancers | Weak Repressed PolyComb \| Enhancers | Weak transcription |
| chr1 | 1093105 | 1093195 |  |  | Enhancers | Active TSS | Flanking Active TSS | Active TSS | Active TSS | Transcr. at gene 5' and 3' |
| chr1 | 1094247 | 1094330 |  |  | Enhancers | Weak Repressed PolyComb | Enhancers | Enhancers | Enhancers | Weak transcription |
| chr1 | 19096151 | 19096229 |  |  | Repressed PolyComb | Weak Repressed PolyComb | Repressed PolyComb | Weak Repressed PolyComb | Weak Repressed PolyComb | Weak Repressed PolyComb |
| chr1 | 21187393 | 21187512 | 362 | JUND | Strong transcription | Strong transcription | Strong transcription | Strong transcription | Weak transcription | Quiescent/Low |
| chr1 | 26753619 | 26753671 |  |  | Weak Repressed PolyComb | Weak Repressed PolyComb | Weak Repressed PolyComb | Quiescent/Low | Quiescent/Low | Weak transcription |
| chr1 | 34907786 | 34907882 |  |  | Weak Repressed PolyComb | Weak Repressed PolyComb | Enhancers | Weak Repressed PolyComb | Weak Repressed PolyComb | Quiescent/Low |
| chr1 | 65296704 | 65296779 |  |  | Weak transcription | Weak transcription | Weak transcription | Weak transcription | Weak transcription | Quiescent/Low |
| chr1 | 68421788 | 68421881 |  |  | Quiescent/Low | Quiescent/Low | Quiescent/Low | Quiescent/Low | Quiescent/Low | Weak Repressed PolyComb |
| chr1 | 94084975 | 94085055 |  |  | Enhancers | Weak transcription | Enhancers | Weak transcription | Weak transcription | Weak transcription |
| chr1 | 100519384 | 100519452 |  |  | Weak transcription | Weak transcription | Weak transcription | Weak transcription | Weak transcription | Weak transcription |
| chr1 | 117015893 | 117015972 |  |  | Genic enhancers | Quiescent/Low | Quiescent/Low | Weak transcription | Weak transcription | Weak transcription |
| chr1 | 149784895 | 149784991 | 323 | POLR2A | Active TSS | Active TSS | Active TSS | Active TSS | Active TSS | Active TSS |
| chr1 | 152432764 | 152432843 | 659, 304 | CTCF, ELF1 | Active TSS | Flanking Active TSS \| Active TSS | Flanking Active TSS \| Active TSS | Weak Repressed PolyComb | Quiescent/Low | Quiescent/Low \| Enhancers |
| chr1 | 153431591 | 153431687 | 152 | HNF4A + Forskolin | Weak Repressed PolyComb | Weak Repressed PolyComb | Quiescent/Low | Weak Repressed PolyComb | Quiescent/Low | Quiescent/Low |
| chr1 | 153582764 | 153582860 |  |  | Flanking Active TSS | Enhancers | Enhancers | Enhancers | Enhancers | Enhancers |
| chr1 | 155172546 | 155172660 |  |  | Repressed PolyComb | Weak transcription | Weak transcription | Quiescent/Low | Weak transcription | Weak transcription |
| chr1 | 170380297 | 170380407 |  |  | Quiescent/Low | Weak Repressed PolyComb | Quiescent/Low | Quiescent/Low | Quiescent/Low | Quiescent/Low |
| chr1 | 200044361 | 200044453 |  |  | Quiescent/Low | Weak transcription \| Enhancers | Weak Repressed PolyComb \| Repressed Polycomb | Weak transcription | Quiescent/Low | Quiescent/Low |
| chr1 | 218358121 | 218358206 |  |  | Quiescent/Low | Weak Repressed PolyComb | Quiescent/Low | Quiescent/Low | Quiescent/Low | Weak Repressed PolyComb |
| chr10 | 14518580 | 14518666 |  |  | Repressed PolyComb | Weak Repressed PolyComb | Weak Repressed PolyComb | Quiescent/Low | Quiescent/Low | Quiescent/Low |
| chr10 | 76982221 | 76982317 |  |  | Genic enhancers | Strong transcription | Strong transcription | Strong transcription | Weak transcription | Weak transcription |
| chr10 | 88014430 | 88014525 |  |  | Weak Repressed PolyComb | Quiescent/Low | Weak Repressed PolyComb | Quiescent/Low | Quiescent/Low | Quiescent/Low |
| chr10 | 91342483 | 91342564 |  |  | Weak transcription | Strong transcription | Quiescent/Low | Weak transcription | Quiescent/Low | Quiescent/Low |
| chr10 | 100144964 | 100145054 |  |  | Quiescent/Low | Strong transcription | Strong transcription \| Genic enhancers | Strong transcription | Strong transcription | Weak transcription |
| chr10 | 102724731 | 102724831 |  |  | Enhancers | Weak transcription | Strong transcription | Weak transcription | Weak transcription | Quiescent/Low |
| chr10 | 105797836 | 105797934 |  |  | Quiescent/Low | Quiescent/Low | Weak Repressed PolyComb | Quiescent/Low | Quiescent/Low | Quiescent/Low |
| chr10 | 105968536 | 105968631 |  |  | Quiescent/Low | Quiescent/Low | Quiescent/Low | Quiescent/Low | Quiescent/Low | Quiescent/Low |
| chr10 | 134911005 | 134911115 |  |  | Repressed PolyComb | Weak Repressed PolyComb | Weak Repressed PolyComb | Weak Repressed PolyComb | Repressed PolyComb | Quiescent/Low |
| chr11 | 558088 | 558198 |  |  | Enhancers | Weak transcription | Repressed PolyComb | Repressed PolyComb | Weak Repressed PolyComb | Weak transcription |
| chr11 | 1974564 | 1974637 | 349, 413, 227 | HNF4G, MYBL2, NFIC | Genic enhancers | Enhancers \| Flanking Active TSS | Genic enhancers | Genic enhancers | Genic enhancers | Weak transcription \| Enhancers |
| chr11 | 2111939 | 2112015 |  |  | Repressed PolyComb | Quiescent/Low | Weak Repressed PolyComb | Quiescent/Low | Weak transcription | Quiescent/Low |
| chr11 | 43559519 | 43559609 |  |  | Repressed PolyComb | Repressed PolyComb | Repressed PolyComb | Repressed PolyComb | Weak Repressed PolyComb | Quiescent/Low |
| chr11 | 61316542 | 61316609 |  |  | Quiescent/Low | Quiescent/Low | Weak Repressed PolyComb | Enhancers | Enhancers | Weak transcription |
| chr11 | 61339208 | 61339288 |  |  | Quiescent/Low | Quiescent/Low | Weak Repressed PolyComb | Enhancers | Enhancers | Enhancers |
| chr11 | 63892649 | 63892751 |  |  | Quiescent/Low | Weak transcription | Quiescent/Low | Weak transcription | Strong transcription | Weak transcription |
| chr11 | 64415184 | 64415294 |  |  | Repressed PolyComb | Weak Repressed PolyComb | Weak Repressed PolyComb | Quiescent/Low | Weak Repressed PolyComb | Weak transcription |
| chr11 | 64415402 | 64415487 |  |  | Repressed PolyComb | Weak Repressed PolyComb | Weak Repressed PolyComb | Quiescent/Low | Weak Repressed PolyComb | Weak transcription |
| chr11 | 72003754 | 72003822 |  |  | Quiescent/Low \| Weak transcription | Strong transcription | Strong transcription | Strong transcription | Weak transcription | Quiescent/Low |
| chr11 | 74723783 | 74723878 |  |  | Enhancers | Strong transcription | Quiescent/Low | Weak transcription | Weak transcription | Quiescent/Low |
| chr11 | 90241936 | 90242018 |  |  | Quiescent/Low | Quiescent/Low | Quiescent/Low | Quiescent/Low | Quiescent/Low | Quiescent/Low |
| chr11 | 93839308 | 93839394 |  |  | Quiescent/Low | Quiescent/Low | Quiescent/Low | Quiescent/Low | Quiescent/Low | Quiescent/Low |
| chr11 | 110888872 | 110888956 |  |  | Weak Repressed PolyComb | Quiescent/Low | Weak transcription | Weak Repressed PolyComb | Quiescent/Low | Quiescent/Low |
| chr11 | 121522439 | 121522511 |  |  | Weak Repressed PolyComb | Weak transcription | Weak Repressed PolyComb | Quiescent/Low | Quiescent/Low | Quiescent/Low |
| chr11 | 121528146 | 121528226 |  |  | Enhancers | Quiescent/Low | Weak transcription | Quiescent/Low | Quiescent/Low | Weak transcription |
| chr12 | 6943122 | 6943190 |  |  | Weak transcription | Quiescent/Low | Weak transcription | Transcr. at gene 5' and 3' | Flanking Active TSS | Active TSS |
| chr12 | 6943520 | 6943615 |  |  | Weak transcription | Quiescent/Low | Weak transcription | Active TSS \| Weak transcription | Weak transcription | Active TSS \| Weak transcription |
| chr12 | 12808849 | 12808944 | 608, 151, 204, 1000, 524 | CTCF, MAFF, MAFK, RAD21, SMC3 | Enhancers | Enhancers | Enhancers | Quiescent/Low | Quiescent/Low | Quiescent/Low |
| chr12 | 12960029 | 12960119 |  |  | Weak transcription | Quiescent/Low | Enhancers | Enhancers | Quiescent/Low | Quiescent/Low |
| chr12 | 52671788 | 52671898 |  |  | Repressed PolyComb | Weak Repressed PolyComb | Repressed PolyComb | Weak Repressed PolyComb | Weak Repressed PolyComb | Weak transcription |
| chr12 | 52714000 | 52714096 |  |  | Repressed PolyComb | Weak Repressed PolyComb | Repressed PolyComb | Weak Repressed PolyComb | Weak Repressed PolyComb | Weak Repressed PolyComb |
| chr12 | 55874553 | 55874626 |  |  | Quiescent/Low | Quiescent/Low | Quiescent/Low | Quiescent/Low | Quiescent/Low | Quiescent/Low |
| chr12 | 56504658 | 56504742 |  |  | Strong transcription | Strong transcription | Strong transcription | Strong transcription | Strong transcription | Quiescent/Low |
| chr12 | 67953203 | 67953265 |  |  | Quiescent/Low | Heterochromatin | Heterochromatin | Quiescent/Low | Quiescent/Low | Quiescent/Low |
| chr12 | 78337167 | 78337232 |  |  | Quiescent/Low | Weak Repressed PolyComb | Enhancers | Quiescent/Low | Quiescent/Low | Quiescent/Low |
| chr12 | 94226326 | 94226420 |  |  | Quiescent/Low | Quiescent/Low | Quiescent/Low | Weak transcription | Weak transcription | Weak transcription |
| chr12 | 99107792 | 99107858 |  |  | Weak transcription | Weak transcription | Weak transcription | Weak transcription | Weak transcription | Weak transcription |
| chr13 | 40282901 | 40282997 |  |  | Quiescent/Low | Weak transcription | Weak transcription | Weak transcription | Weak transcription | Quiescent/Low |
| chr13 | 49521255 | 49521338 |  |  | Quiescent/Low | Quiescent/Low | Quiescent/Low | Quiescent/Low | Quiescent/Low | Quiescent/Low |
| chr13 | 90800859 | 90800943 |  |  | Quiescent/Low | Quiescent/Low | Quiescent/Low | Quiescent/Low | Quiescent/Low | Quiescent/Low |
| chr13 | 90801099 | 90801260 |  |  | Quiescent/Low | Quiescent/Low | Quiescent/Low | Quiescent/Low | Quiescent/Low | Quiescent/Low |
| chr13 | 90801319 | 90801390 |  |  | Quiescent/Low | Quiescent/Low | Quiescent/Low | Quiescent/Low | Quiescent/Low | Quiescent/Low |
| chr13 | 90801446 | 90801533 |  |  | Quiescent/Low | Quiescent/Low | Quiescent/Low | Quiescent/Low | Quiescent/Low | Quiescent/Low |
| chr13 | 90801568 | 90801646 |  |  | Quiescent/Low | Quiescent/Low | Quiescent/Low | Quiescent/Low | Quiescent/Low | Quiescent/Low |
| chr14 | 22927644 | 22927715 |  |  | Weak Repressed PolyComb | Weak Repressed PolyComb | Weak transcription | Quiescent/Low | Quiescent/Low | Quiescent/Low |
| chr14 | 22957035 | 22957112 |  |  | Repressed PolyComb | Weak Repressed PolyComb | Weak Repressed PolyComb | Enhancers | Enhancers | Enhancers |
| chr14 | 30553602 | 30553699 |  |  | Heterochromatin | Quiescent/Low | Quiescent/Low | Quiescent/Low | Quiescent/Low | Quiescent/Low |
| chr14 | 65007572 | 65007657 | 238, 201 | MYBL2, TBP | Active TSS \| Quiescent/Low | Flanking Active TSS\| Flanking Bivalent TSS/Enh | Flanking Active TSS \| Flanking Bivalent TSS/Enh | Active TSS \| Flanking active TSS | Flanking Active TSS | Active TSS |
| chr14 | 100405149 | 100405238 |  |  | Quiescent/Low | Weak transcription | Strong transcription | Strong transcription | Strong transcription | Weak transcription |
| chr14 | 100410582 | 100410675 |  |  | Flanking Active TSS | Flanking Active TSS | Weak transcription | Weak transcription | Weak transcription | Weak transcription \| Enhancers |
| chr14 | 100411122 | 100411194 |  |  | Enhancers | Enhancers | Weak transcription | Weak transcription | Weak transcription | Quiescent/Low |
| chr14 | 100417096 | 100417210 |  |  | Bivalent Enhancer \| Weak Repressed PolyComb | Quiescent/Low | Weak Repressed PolyComb | Quiescent/Low | Weak Repressed PolyComb | Quiescent/Low |
| chr14 | 100559414 | 100559510 | 216, 197, 193 | FOSL2, FOXA1, FOXA2 | Weak transcription | Weak transcription | Weak transcription | Weak transcription | Weak transcription | Quiescent/Low |
| chr14 | 100559883 | 100559946 |  |  | Weak transcription | Weak transcription | Weak transcription | Weak transcription | Weak transcription | Quiescent/Low |
| chr14 | 100563189 | 100563273 |  |  | Strong transcription | Strong transcription | Weak transcription | Weak transcription | Weak transcription | Quiescent/Low |
| chr14 | 100576871 | 100576939 |  |  | Weak transcription | Weak transcription | Weak transcription | Weak transcription | Weak transcription | Weak transcription |
| chr14 | 100580287 | 100580373 |  |  | Weak transcription | Weak transcription | Weak transcription | Weak transcription | Weak transcription | Weak transcription |
| chr14 | 100582009 | 100582084 |  |  | Weak transcription | Weak transcription | Weak transcription | Weak transcription | Weak transcription | Weak transcription |
| chr14 | 100582544 | 100582628 |  |  | Weak transcription | Weak transcription | Weak transcription | Weak transcription | Weak transcription | Weak transcription |
| chr14 | 100583410 | 100583488 |  |  | Weak transcription | Strong transcription | Weak transcription | Weak transcription | Weak transcription | Weak transcription |
| chr14 | 100585639 | 100585736 |  |  | Weak transcription | Weak transcription | Weak transcription | Weak transcription | Weak transcription | Weak transcription |
| chr14 | 100588535 | 100588615 |  |  | Weak transcription | Weak transcription | Weak transcription | Weak transcription | Weak transcription | Weak transcription |
| chr14 | 100590395 | 100590471 |  |  | Weak transcription | Strong transcription | Strong transcription | Weak transcription | Strong transcription | Weak transcription |
| chr14 | 100595844 | 100595928 |  |  | Weak transcription | Strong transcription | Enhancers | Enhancers | Genic enhancers | Enhancers |
| chr14 | 100596662 | 100596764 |  |  | Weak transcription | Strong transcription | Enhancers | Weak transcription | Strong transcription | Quiescent/Low |
| chr14 | 100600584 | 100600668 |  |  | Enhancers | Strong transcription | Genic enhancers | Strong transcription | Strong transcription | Quiescent/Low |
| chr14 | 100601389 | 100601468 |  |  | Weak transcription | Strong transcription | Strong transcription | Weak transcription | Strong transcription | Quiescent/Low |
| chr14 | 100601536 | 100601627 |  |  | Weak transcription | Strong transcription | Strong transcription | Weak transcription | Strong transcription \| Weak transcription | Quiescent/Low |
| chr14 | 100601687 | 100601757 |  |  | Weak transcription | Strong transcription | Strong transcription | Weak transcription | Weak transcription | Quiescent/Low |
| chr14 | 100602001 | 100602081 |  |  | Weak transcription | Strong transcription | Strong transcription | Weak transcription | Weak transcription | Quiescent/Low |
| chr14 | 100602813 | 100602891 |  |  | Weak transcription | Strong transcription | Strong transcription | Weak transcription | Weak transcription | Quiescent/Low |
| chr15 | 29144526 | 29144636 |  |  | Quiescent/Low | Quiescent/Low | Weak Repressed PolyComb | Weak Repressed PolyComb | Weak Repressed PolyComb | Weak transcription |
| chr15 | 43512539 | 43512619 |  |  | Weak Repressed PolyComb | Quiescent/Low | Enhancers \| Quiescent/Low | Quiescent/Low | Quiescent/Low | Weak transcription |
| chr15 | 50356605 | 50356689 |  |  | Quiescent/Low | Quiescent/Low | Quiescent/Low | Quiescent/Low | Quiescent/Low | Quiescent/Low |
| chr15 | 53452429 | 53452524 | 268 | RAD21 | Enhancers | Flanking Active TSS | Quiescent/Low | Quiescent/Low | Quiescent/Low | Weak Repressed PolyComb |
| chr15 | 61950181 | 61950271 |  |  | Weak Repressed PolyComb | Quiescent/Low | Weak transcription | Weak Repressed PolyComb | Quiescent/Low | Weak transcription |
| chr15 | 68158764 | 68158861 |  |  | Quiescent/Low | Quiescent/Low | Quiescent/Low | Quiescent/Low | Quiescent/Low | Quiescent/Low |
| chr15 | 70666611 | 70666708 |  |  | Weak Repressed PolyComb | Quiescent/Low | Enhancers | Quiescent/Low | Enhancers | Quiescent/Low |
| chr15 | 77289184 | 77289268 |  |  | Repressed PolyComb \| Weak Repressed PolyComb | Active TSS | Flanking Active TSS | Flanking Active TSS \| Enhancers | Enhancers | Enhancers |
| chr15 | 84114730 | 84114813 |  |  | Repressed PolyComb | Weak Repressed PolyComb | Weak Repressed PolyComb | Weak Repressed PolyComb | Quiescent/Low | Weak Repressed PolyComb |
| chr15 | 86952341 | 86952432 |  |  | Heterochromatin | Weak Repressed PolyComb | Quiescent/Low | Weak Repressed PolyComb \| Heterochromatin | Quiescent/Low | Quiescent/Low |
| chr15 | 87712251 | 87712341 |  |  | Heterochromatin | Quiescent/Low | Quiescent/Low | Quiescent/Low | Quiescent/Low | Quiescent/Low |
| chr15 | 94677493 | 94677540 |  |  | Quiescent/Low | Repressed PolyComb | Weak Repressed PolyComb | Weak Repressed PolyComb | Quiescent/Low | Quiescent/Low |
| chr15 | 100318184 | 100318322 |  |  | Quiescent/Low | Quiescent/Low | Quiescent/Low | Quiescent/Low | Quiescent/Low | Quiescent/Low |
| chr16 | 760183 | 760278 | 132 | CTCF | Repressed PolyComb | Weak Repressed PolyComb | Repressed PolyComb | Weak Repressed PolyComb | Enhancers \| Weak Repressed Polycomb | Weak transcription |
| chr16 | 2080196 | 2080286 |  |  | Weak transcription | Flanking Active TSS | Transcr. at gene 5' and 3' | Active TSS | Active TSS | Active TSS |
| chr16 | 2261748 | 2261842 |  |  | Weak transcription | Weak transcription | Weak transcription | Enhancers | Weak transcription \| Enhancers | Weak transcription \| Enhancers |
| chr16 | 14305324 | 14305407 |  |  | Weak transcription | Weak transcription | Weak transcription \| Strong transcription | Weak transcription | Weak transcription | Enhancers |
| chr16 | 15644651 | 15644730 | 510, 186, 154, 128 | BHLHE40, RCOR1, RFX5, YY1 | Enhancers | Weak transcription | Weak transcription | Weak transcription | Quiescent/Low | Quiescent/Low |
| chr16 | 68157211 | 68157272 |  |  | Strong transcription | Weak transcription | Weak transcription | Weak transcription | Weak transcription | Weak transcription |
| chr16 | 68524484 | 68524584 |  |  | Weak Repressed PolyComb | Weak transcription | Weak Repressed PolyComb | Quiescent/Low | Weak Repressed PolyComb | Weak transcription |
| chr17 | 1563946 | 1564031 |  |  | Strong transcription | Strong transcription | Strong transcription | Strong transcription | Strong transcription | Weak transcription |
| chr17 | 1899951 | 1900052 | 327, 302 | FOXA1, FOXA2 | Flanking Active TSS | Flanking Active TSS | Quiescent/Low | Enhancers | Weak transcription | Enhancers |
| chr17 | 2598121 | 2598226 |  |  | Weak transcription | Strong transcription | Strong transcription | Strong transcription | Strong transcription | Weak transcription |
| chr17 | 17657874 | 17657970 |  |  | Weak transcription | Weak transcription | Weak transcription | Enhancers | Enhancers | Weak transcription |
| chr17 | 19188411 | 19188480 |  |  | Weak transcription | Weak transcription | Weak transcription | Enhancers | Enhancers | Enhancers |
| chr17 | 26911127 | 26911215 |  |  | Strong transcription | Strong transcription | Strong transcription | Weak transcription | Strong transcription | Weak transcription |
| chr17 | 44012198 | 44012308 |  |  | Flanking Active TSS | Flanking Active TSS | Quiescent/Low | Weak transcription | Enhancers \| Weak transcription | Quiescent/Low |
| chr17 | 44064850 | 44064920 |  |  | Bivalent Enhancer | Quiescent/Low | Weak transcription | Quiescent/Low | Weak transcription | Weak transcription |
| chr17 | 54569900 | 54570015 |  |  | Weak Repressed PolyComb | Weak Repressed PolyComb | Quiescent/Low | Weak Repressed PolyComb | Quiescent/Low | Weak Repressed PolyComb |
| chr17 | 54583278 | 54583364 | 182, 394 | MAFF, MAFK | Weak Repressed PolyComb | Weak Repressed PolyComb | Quiescent/Low | Weak Repressed PolyComb | Weak Repressed PolyComb | Quiescent/Low |
| chr17 | 72244126 | 72244225 |  |  | Quiescent/Low | Quiescent/Low | Weak transcription | Weak transcription | Strong transcription | Weak transcription |
| chr17 | 76713670 | 76713768 |  |  | Flanking Active TSS | Flanking Active TSS | Flanking Active TSS | Flanking Active TSS | Flanking Active TSS | Active TSS |
| chr17 | 76714277 | 76714344 | 182, 271, 264, 313, 512, 229, 597, 166, 238, 154, 212 | BHLHE40, EP300, FOSL2, FOXA1, FOXA2, MXI1, NFIC, RCOR1, RXRA, SIN3AK2, SP1 | Enhancers | Enhancers | Enhancers | Enhancers | Enhancers | Enhancers |
| chr17 | 76721590 | 76721703 |  |  | Enhancers | Enhancers | Enhancers | Enhancers | Enhancers | Enhancers |
| chr18 | 31738778 | 31738887 |  |  | Repressed PolyComb | Repressed PolyComb \| Bivalent Enhancer | Repressed PolyComb \| Flanking Bivalent TSS/Enh | Weak Repressed PolyComb \| Repressed PolyComb | Weak Repressed PolyComb \| Bivalent Enhancer | Weak Repressed PolyComb \| Flanking Active TSS |
| chr18 | 45267740 | 45267790 |  |  | Weak Repressed PolyComb | Quiescent/Low | Weak Repressed PolyComb | Weak Repressed PolyComb | Weak Repressed PolyComb | Quiescent/Low |
| chr18 | 54269249 | 54269400 |  |  | Weak transcription | Strong transcription | Strong transcription | Weak transcription | Weak transcription | Quiescent/Low |
| chr19 | 1767157 | 1767237 |  |  | Repressed PolyComb | Weak Repressed PolyComb | Repressed PolyComb | Repressed PolyComb | Repressed PolyComb | Weak transcription |
| chr19 | 2185060 | 2185148 |  |  | Strong transcription | Strong transcription | Weak transcription | Enhancers | Enhancers | Enhancers |
| chr19 | 10789101 | 10789172 |  |  | Strong transcription | Genic enhancers | Strong transcription | Strong transcription | Strong transcription | Weak transcription |
| chr19 | 45480289 | 45480388 |  |  | Strong transcription | Strong transcription | Strong transcription | Strong transcription | Strong transcription | Weak transcription |
| chr19 | 50834091 | 50834185 | 198, 200, 127, 292 | MAZ, MXI1, SP1, TAF1 | Active TSS | Enhancers | Bivalent Enhancer | Active TSS | Active TSS | Transcr. at gene 5' and 3' |
| chr19 | 54695853 | 54695937 |  |  | Flanking Active TSS | Flanking Active TSS | Flanking Active TSS | Enhancers | Flanking Active TSS | Transcr. at gene 5' and 3' |
| chr19 | 56887676 | 56887746 |  |  | Heterochromatin | ZNF genes & repeats | ZNF genes & repeats | Strong transcription | Strong transcription | Quiescent/Low |
| chr19 | 56888318 | 56888404 |  |  | ZNF genes & repeats | ZNF genes & repeats | ZNF genes & repeats | Strong transcription \| ZNF genes & repeats | Strong transcription | Quiescent/Low \| Heterochromatin |
| chr19 | 58861744 | 58861828 |  |  | Weak transcription | Flanking Active TSS | Bivalent Enhancer | Bivalent Enhancer | Flanking Active TSS | Weak transcription \| Enhancers |
| chr19 | 58864222 | 58864320 |  |  | Flanking Active TSS | Flanking Active TSS | Repressed PolyComb | Weak Repressed PolyComb | Weak Repressed PolyComb | Weak transcription |
| chr19 | 58867033 | 58867106 |  |  | Weak transcription | Enhancers | Repressed PolyComb | Weak Repressed PolyComb | Enhancers | Weak transcription |
| chr19 | 58869262 | 58869386 |  |  | Weak transcription | Weak transcription | Weak Repressed PolyComb | Enhancers | Enhancers | Enhancers |
| chr19 | 58870776 | 58870863 |  |  | Weak transcription | Weak transcription | Weak Repressed PolyComb | Weak transcription | Weak transcription | Weak transcription |
| chr19 | 58874068 | 58874151 | 154, 141, 451, 155, 1000, 222, 117, 261, 166, 873, 332, 169 | CEBPB + Forskolin, CEBPD, HSF1 + Forskolin, MAX, MXI1, NRF1, PGC1A, POLR2A, REST, SIN3AK2, TAF1, TBP | Active TSS | Active TSS | Active TSS | Active TSS | Active TSS | Transcr. at gene 5' and 3' |
| chr19 | 58880074 | 58880157 |  |  | Quiescent/Low | Weak transcription | Quiescent/Low | Weak Repressed PolyComb | Enhancers | Quiescent/Low |
| chr19 | 58889458 | 58889541 |  |  | Weak transcription | Weak transcription | Quiescent/Low | Quiescent/Low | Weak transcription | Quiescent/Low |
| chr19 | 58892598 | 58892683 | 1000, MAX, MAZ, MXI1, RFX5, SIN3AK2, SP1, SREBP1 | ELF1, MAX, MAZ, MXI1, RFX5, SIN3AK2, SP1, SREBP1 | Active TSS | Active TSS \| Flanking Active TSS | Active TSS | Active TSS | Active TSS | Active TSS |
| chr19 | 58893450 | 58893537 |  |  | Enhancers | Enhancers | Flanking Active TSS | Enhancers | Enhancers | Weak transcription |
| chr19 | 58896292 | 58896353 |  |  | Enhancers | Weak transcription | Weak transcription | Weak transcription | Weak transcription | Weak transcription |
| chr19 | 58897802 | 58897885 |  |  | Active TSS | Flanking Active TSS | Flanking Active TSS | Active TSS | Active TSS | Transcr. at gene 5' and 3' |
| chr19 | 58906067 | 58906154 | 194, 225 | MAFF, MAFK | Strong transcription | Strong transcription | Strong transcription | Strong transcription | Strong transcription | Weak transcription |
| chr19 | 58907333 | 58907420 |  |  | Weak transcription | Bivalent Enhancer | Bivalent Enhancer | Weak transcription \| Bivalent Enhancer | Flanking Active TSS | Weak transcription |
| chr19 | 58908412 | 58908500 |  |  | Weak transcription | Weak Repressed PolyComb | Repressed PolyComb | Weak Repressed PolyComb | Weak Repressed PolyComb | Weak transcription |
| chr19 | 58911659 | 58911746 |  |  | Flanking Active TSS | Weak Repressed PolyComb | Weak Repressed PolyComb | Weak Repressed PolyComb | Enhancers | Weak transcription |
| chr19 | 58920507 | 58920592 |  |  | Active TSS | Active TSS | Flanking Active TSS | Active TSS | Active TSS | Transcr. at gene 5' and 3' |
| chr19 | 58937577 | 58937665 |  |  | Quiescent/Low | ZNF genes & repeats | Heterochromatin | ZNF genes & repeats | Weak transcription | Quiescent/Low \| Heterochromatin |
| chr19 | 58943701 | 58943788 |  |  | Quiescent/Low | ZNF genes & repeats | ZNF genes & repeats | ZNF genes & repeats | ZNF genes & repeats | Heterochromatin |
| chr19 | 58946276 | 58946363 |  |  | Quiescent/Low | ZNF genes & repeats | ZNF genes & repeats | ZNF genes & repeats | ZNF genes & repeats | ZNF genes & repeats |
| chr19 | 58949083 | 58949168 |  |  | Quiescent/Low | ZNF genes & repeats | ZNF genes & repeats | ZNF genes & repeats | Weak transcription | Heterochromatin |
| chr19 | 58951806 | 58951896 |  |  | Quiescent/Low | Active TSS | Flanking Active TSS | Active TSS | Active TSS | Active TSS |
| chr19 | 58953297 | 58953384 |  |  | Quiescent/Low | Heterochromatin | Weak Repressed PolyComb | Quiescent/Low | Quiescent/Low | Weak transcription |
| chr19 | 58956198 | 58956288 |  |  | Quiescent/Low | Heterochromatin | Weak Repressed PolyComb | Quiescent/Low | Quiescent/Low | Weak transcription |
| chr19 | 58982955 | 58983022 |  |  | Weak transcription | Strong transcription | Strong transcription | Strong transcription | Strong transcription | Weak transcription |
| chr19 | 58983770 | 58983839 |  |  | Strong transcription | Strong transcription | Strong transcription | Strong transcription | Strong transcription | Weak transcription |
| chr2 | 56081352 | 56081434 |  |  | Quiescent/Low | Quiescent/Low | Quiescent/Low | Quiescent/Low | Quiescent/Low | Quiescent/Low |
| chr2 | 114057005 | 114057143 |  |  | Quiescent/Low | Quiescent/Low | Weak Repressed PolyComb | Quiescent/Low | Quiescent/Low | Quiescent/Low |
| chr2 | 175740606 | 175740683 |  |  | Quiescent/Low | Quiescent/Low | Quiescent/Low | Weak transcription | Weak transcription | Quiescent/Low |
| chr2 | 219574610 | 219574674 |  |  | Weak transcription | Weak transcription | Weak transcription | Weak transcription | Weak transcription | Quiescent/Low |
| chr2 | 219867076 | 219867166 | 281, 233, 211 | MXI1, NFIC, TAF1 | Flanking Bivalent TSS/Enh | Flanking Active TSS | Bivalent Enhancer | Bivalent Enhancer | Flanking Active TSS | Genic enhancers |
| chr2 | 232465195 | 232465252 |  |  | Weak Repressed PolyComb | Quiescent/Low | Weak transcription | Quiescent/Low | Weak transcription | Weak transcription |
| chr2 | 232745606 | 232745701 |  |  | Weak Repressed PolyComb | Weak Repressed PolyComb | Weak Repressed PolyComb | Weak Repressed PolyComb | Weak Repressed PolyComb | Weak Repressed PolyComb |
| chr2 | 241044090 | 241044179 |  |  | Weak Repressed PolyComb | Weak Repressed PolyComb | Weak Repressed PolyComb | Weak Repressed PolyComb | Weak Repressed PolyComb | Weak Repressed PolyComb |
| chr20 | 30289258 | 30289311 |  |  | Genic enhancers | Weak transcription | Weak transcription | Weak transcription | Weak transcription | Weak transcription |
| chr20 | 33505189 | 33505333 |  |  | Weak transcription | Strong transcription | Strong transcription | Weak transcription | Weak transcription | Weak transcription |
| chr20 | 56826064 | 56826144 |  |  | Quiescent/Low | Weak transcription | Strong transcription | Strong transcription | Strong transcription | Quiescent/Low |
| chr20 | 56826675 | 56826763 |  |  | Quiescent/Low | Weak transcription | Weak transcription | Strong transcription | Strong transcription | Quiescent/Low |
| chr20 | 59961996 | 59962113 |  |  | Repressed PolyComb | Weak Repressed PolyComb | Weak Repressed PolyComb | Weak Repressed PolyComb | Weak Repressed PolyComb | Quiescent/Low |
| chr20 | 60572563 | 60572665 |  |  | Genic enhancers | Strong transcription | Strong transcription | Strong transcription | Strong transcription | Weak transcription |
| chr20 | 62043261 | 62043341 | 329 | GABPA | Flanking Bivalent TSS/Enh | Quiescent/Low | Repressed PolyComb | Weak Repressed PolyComb | Weak Repressed PolyComb | Weak transcription |
| chr20 | 62044427 | 62044523 |  |  | Repressed PolyComb | Quiescent/Low | Repressed PolyComb | Weak Repressed PolyComb | Weak Repressed PolyComb | Weak transcription |
| chr21 | 16834018 | 16834102 |  |  | Enhancers | Quiescent/Low | Quiescent/Low | Quiescent/Low | Quiescent/Low | Quiescent/Low |
| chr21 | 16884427 | 16884516 |  |  | Weak transcription | Quiescent/Low | Quiescent/Low | Quiescent/Low | Quiescent/Low | Weak Repressed PolyComb |
| chr21 | 25868162 | 25868227 |  |  | Heterochromatin | Quiescent/Low | Quiescent/Low | Quiescent/Low | Quiescent/Low | Quiescent/Low |
| chr21 | 36014882 | 36014976 |  |  | Weak Repressed PolyComb | Weak Repressed PolyComb | Weak Repressed PolyComb | Quiescent/Low | Quiescent/Low | Quiescent/Low |
| chr22 | 18400661 | 18400743 |  |  | Enhancers | Weak transcription | Weak transcription | Enhancers | Enhancers | Genic enhancers |
| chr22 | 18616656 | 18616734 |  |  | Weak transcription | Enhancers | Enhancers | Weak transcription | Weak transcription | Quiescent/Low |
| chr22 | 19718464 | 19718561 | 216, 589, 171, 184, 252, 328, 179, 139, 1000, 139, 1000, 139, 161, 373 | CEBPD, CTCF + Forskolin, ELF1, EP300, GABPA, HDAC2, HNF4G, MAZ, RAD21, RXRA, SIN3AK2, SMC3 | Active TSS | Enhancers | Flanking Active TSS | Enhancers | Flanking Active TSS | Transcr. at gene 5' and 3' |
| chr22 | 36570224 | 36570324 |  |  | Weak Repressed PolyComb | Quiescent/Low | Quiescent/Low | Quiescent/Low | Quiescent/Low | Quiescent/Low |
| chr22 | 36573630 | 36573727 |  |  | Weak Repressed PolyComb | Quiescent/Low | Quiescent/Low | Quiescent/Low | Quiescent/Low | Quiescent/Low |
| chr22 | 43975498 | 43975564 |  |  | Weak Repressed PolyComb | Quiescent/Low | Weak Repressed PolyComb | Weak Repressed PolyComb | Quiescent/Low | Quiescent/Low |
| chr22 | 44887292 | 44887366 |  |  | Repressed PolyComb | Weak transcription | Weak transcription | Weak transcription | Weak transcription | Quiescent/Low |
| chr3 | 10411172 | 10411246 |  |  | Weak Repressed PolyComb | Strong transcription | Weak Repressed PolyComb | Quiescent/Low | Quiescent/Low | Weak transcription |
| chr3 | 35760971 | 35761055 |  |  | Quiescent/Low | Quiescent/Low | Quiescent/Low | Quiescent/Low | Quiescent/Low | Quiescent/Low |
| chr3 | 47866048 | 47866123 |  |  | Flanking Active TSS | Enhancers | Flanking Active TSS | Enhancers | Flanking Active TSS | Enhancers |
| chr3 | 185441886 | 185441971 |  |  | Enhancers | Quiescent/Low | Quiescent/Low | Enhancers | Quiescent/Low | Enhancers |
| chr3 | 187987154 | 187987260 |  |  | Enhancers | Enhancers | Weak transcription | Weak transcription \| Enhancers | Weak transcription | Weak transcription |
| chr3 | 191030404 | 191030492 |  |  | Quiescent/Low | Quiescent/Low | Quiescent/Low | Quiescent/Low | Quiescent/Low | Quiescent/Low |
| chr4 | 1957908 | 1958002 |  |  | Strong transcription | Strong transcription | Strong transcription | Strong transcription | Strong transcription | Quiescent/Low |
| chr4 | 9166886 | 9167035 |  |  | Weak Repressed PolyComb | Quiescent/Low | Quiescent/Low | Quiescent/Low | Quiescent/Low | Quiescent/Low |
| chr4 | 10979548 | 10979643 |  |  | Quiescent/Low | Quiescent/Low | Quiescent/Low | Weak Repressed PolyComb | Quiescent/Low | Quiescent/Low |
| chr4 | 24130912 | 24131011 |  |  | Quiescent/Low | Quiescent/Low | Quiescent/Low | Quiescent/Low | Quiescent/Low | Quiescent/Low |
| chr4 | 36104382 | 36104445 |  |  | Quiescent/Low | Weak transcription | Quiescent/Low | Quiescent/Low | Quiescent/Low | Quiescent/Low |
| chr4 | 110629302 | 110629400 |  |  | Weak transcription | Strong transcription | Weak transcription | Weak transcription | Weak transcription | Quiescent/Low |
| chr4 | 115797363 | 115797459 |  |  | Quiescent/Low | Quiescent/Low | Quiescent/Low | Quiescent/Low | Quiescent/Low | Quiescent/Low |
| chr4 | 148485230 | 148485319 |  |  | Quiescent/Low | Heterochromatin | Quiescent/Low | Quiescent/Low | Quiescent/Low | Quiescent/Low |
| chr5 | 36183750 | 36183847 |  |  | Weak transcription | Weak transcription | Strong transcription | Weak transcription | Weak transcription | Quiescent/Low |
| chr5 | 53283090 | 53283186 |  |  | Quiescent/Low | Quiescent/Low | Quiescent/Low | Quiescent/Low | Quiescent/Low | Quiescent/Low |
| chr5 | 87998426 | 87998513 |  |  | Quiescent/Low | Weak Repressed PolyComb | Weak Repressed PolyComb | Weak Repressed PolyComb | Quiescent/Low | Weak Repressed PolyComb |
| chr5 | 100180084 | 100180168 |  |  | Quiescent/Low | Quiescent/Low | Quiescent/Low | Quiescent/Low | Quiescent/Low | Quiescent/Low |
| chr5 | 132791186 | 132791297 |  |  | Weak Repressed PolyComb | Enhancers | Quiescent/Low | Weak Repressed PolyComb | Weak Repressed PolyComb | Weak transcription |
| chr5 | 148422068 | 148422165 |  |  | Quiescent/Low | Quiescent/Low | Quiescent/Low | Weak transcription | Quiescent/Low | Quiescent/Low |
| chr5 | 148790401 | 148790489 | 226, 169, 165 | HNF4A, HNF4G, RFX5 | Flanking Active TSS | Quiescent/Low | Flanking Active TSS | Active TSS | Active TSS | Active TSS |
| chr5 | 154045528 | 154045614 |  |  | Quiescent/Low | Quiescent/Low | Heterochromatin | Heterochromatin | Heterochromatin \| Quiescent/Low | Heterochromatin |
| chr5 | 168127728 | 168127838 |  |  | Weak Repressed PolyComb\| Repressed Polycomb | Quiescent/Low | Strong transcription | Quiescent/Low | Weak transcription | Weak transcription |
| chr5 | 168623182 | 168623276 |  |  | Quiescent/Low | Weak Repressed PolyComb | Weak transcription | Quiescent/Low | Quiescent/Low | Weak transcription |
| chr5 | 179157883 | 179157952 |  |  | Genic enhancers | Genic enhancers | Strong transcription | Weak transcription | Weak transcription | Weak transcription |
| chr6 | 32032594 | 32032696 |  |  | Repressed PolyComb | Quiescent/Low | Weak transcription | Weak transcription | Weak transcription | Weak transcription |
| chr6 | 34075726 | 34075806 |  |  | Repressed PolyComb | Weak Repressed PolyComb | Weak Repressed PolyComb | Weak Repressed PolyComb | Repressed PolyComb | Weak transcription |
| chr6 | 45273388 | 45273485 |  |  | Quiescent/Low | Quiescent/Low | Quiescent/Low | Quiescent/Low | Quiescent/Low | Quiescent/Low |
| chr6 | 52117105 | 52117191 |  |  | Weak Repressed PolyComb | Quiescent/Low | Weak Repressed PolyComb | Weak Repressed PolyComb | Weak Repressed PolyComb | Weak Repressed PolyComb |
| chr6 | 119431910 | 119432007 | 413, 315, 1000, 643 | CTCF + Forskolin, CTCF, RAD21, SMC3 | Flanking Active TSS \| Active TSS | Quiescent/Low | Quiescent/Low | Quiescent/Low | Quiescent/Low | Quiescent/Low |
| chr6 | 126847469 | 126847552 |  |  | Quiescent/Low | Quiescent/Low | Quiescent/Low | Quiescent/Low | Quiescent/Low | Quiescent/Low |
| chr6 | 135601990 | 135602087 |  |  | Quiescent/Low | Quiescent/Low | Enhancers | Quiescent/Low | Quiescent/Low | Quiescent/Low |
| chr7 | 18133367 | 18133457 |  |  | Quiescent/Low | Quiescent/Low | Quiescent/Low | Quiescent/Low | Quiescent/Low | Quiescent/Low |
| chr7 | 25956063 | 25956131 |  |  | Weak Repressed PolyComb | Quiescent/Low | Weak Repressed PolyComb | Repressed PolyComb | Weak Repressed PolyComb | Weak Repressed PolyComb |
| chr7 | 27175623 | 27175707 | 460, 196, 517, 680, 641, 265, 224, 469, 212, 302, 305, 193, 816, 163, 177 | ARID3A, BHLHE40, EP300, FOSL2, FOXA2, HNF4G, JUN, JUND, MAFF, MYBL2, NFIC, RAD21, RXRA, SIN3AK2, SP1 | Flanking Active TSS | Repressed PolyComb | Enhancers | Repressed PolyComb | Weak Repressed PolyComb | Weak Repressed PolyComb |
| chr7 | 36925486 | 36925562 |  |  | Weak Repressed PolyComb | Strong transcription | Strong transcription | Weak transcription | Weak transcription | Quiescent/Low |
| chr7 | 73243463 | 73243560 |  |  | Flanking Active TSS | Quiescent/Low | Weak transcription | Quiescent/Low | Weak Repressed PolyComb | Weak transcription |
| chr7 | 91671264 | 91671348 | 115, 353, 286, 185, 433, 146, 133, 188, 128, 117 | CEBPD, EP300, HDAC2, JUND, MYBL2, NRF1, RAD21, RCOR1, SMC3, TBP | Flanking Active TSS | Enhancers | Strong transcription | Weak transcription | Weak transcription | Quiescent/Low |
| chr7 | 92950007 | 92950103 |  |  | Weak transcription | Strong transcription | Strong transcription | ZNF genes & repeats | Quiescent/Low | Quiescent/Low |
| chr7 | 92951183 | 92951267 |  |  | Weak transcription | Strong transcription \| Weak transcription | Strong transcription | Weak transcription | Quiescent/Low | Quiescent/Low |
| chr7 | 95686909 | 95687004 |  |  | Weak Repressed PolyComb | Quiescent/Low | Quiescent/Low | Weak transcription | Quiescent/Low | Weak Repressed PolyComb |
| chr7 | 101833193 | 101833307 |  |  | Strong transcription | Strong transcription | Strong transcription | Strong transcription | Weak transcription | Weak transcription |
| chr7 | 126485377 | 126485474 |  |  | Quiescent/Low | Quiescent/Low | Quiescent/Low | Quiescent/Low | Quiescent/Low | Quiescent/Low |
| chr7 | 129201767 | 129201845 |  |  | Flanking Bivalent TSS/Enh \| Bivalent Enhancer | Quiescent/Low | Weak Repressed PolyComb | Weak Repressed PolyComb | Weak Repressed PolyComb | Enhancers \| Quiescent/Low |
| chr7 | 136238453 | 136238581 |  |  | Heterochromatin | Quiescent/Low | Quiescent/Low | Quiescent/Low | Quiescent/Low | Quiescent/Low |
| chr8 | 1752803 | 1752880 |  |  | Weak Repressed PolyComb | Quiescent/Low | Quiescent/Low | Weak transcription | Quiescent/Low | Quiescent/Low |
| chr8 | 9636591 | 9636688 |  |  | Weak transcription | Strong transcription \| Weak transcription | Strong transcription | Weak transcription | Strong transcription \| Weak transcription | Quiescent/Low |
| chr8 | 9798307 | 9798392 |  |  | Weak Repressed PolyComb | Weak Repressed PolyComb | Weak Repressed PolyComb | Quiescent/Low | Heterochromatin | Weak Repressed PolyComb |
| chr8 | 10930125 | 10930222 |  |  | Weak Repressed PolyComb | Weak Repressed PolyComb | Weak Repressed PolyComb | Weak Repressed PolyComb | Weak Repressed PolyComb | Weak transcription |
| chr8 | 14755317 | 14755390 |  |  | Quiescent/Low | Quiescent/Low | Quiescent/Low | Quiescent/Low | Quiescent/Low | Quiescent/Low |
| chr8 | 26962286 | 26962397 |  |  | Quiescent/Low | Quiescent/Low | Quiescent/Low | Quiescent/Low | Quiescent/Low | Quiescent/Low |
| chr8 | 113724897 | 113724988 |  |  | Quiescent/Low | Quiescent/Low | Quiescent/Low | Quiescent/Low | Quiescent/Low | Quiescent/Low |
| chr8 | 124429454 | 124429551 | 133 | YY1 | Bivalent/Poised TSS | Flanking Active TSS | Flanking Active TSS | Active TSS | Active TSS | Active TSS |
| chr8 | 128877389 | 128877456 |  |  | Genic enhancers | Quiescent/Low | Quiescent/Low | Weak transcription | Weak transcription | Weak transcription |
| chr8 | 129231543 | 129231616 |  |  | Quiescent/Low | Quiescent/Low | Quiescent/Low | Quiescent/Low | Quiescent/Low | Quiescent/Low |
| chr8 | 145596283 | 145596367 |  |  | Flanking Active TSS | Weak transcription | Enhancers | Weak transcription | Weak transcription | Quiescent/Low |
| chr9 | 4840296 | 4840375 | 417, 898 | MAFF, MAFK | Flanking Active TSS | Active TSS | Strong transcription | Weak transcription | Weak transcription | Quiescent/Low |
| chr9 | 21502113 | 21502184 |  |  | Quiescent/Low | Weak Repressed PolyComb | Quiescent/Low | Weak Repressed PolyComb | Quiescent/Low | Quiescent/Low |
| chr9 | 95980936 | 95981023 |  |  | Weak transcription | Quiescent/Low | Weak Repressed PolyComb | Enhancers | Enhancers | Weak transcription |
| chr9 | 110848329 | 110848399 |  |  | Quiescent/Low | Quiescent/Low | Quiescent/Low | Quiescent/Low | Quiescent/Low | Quiescent/Low |
| chr9 | 138684874 | 138684959 | 120, 269, 227 | CTCF + Forskolin, CTCF, RAD21 | Repressed PolyComb | Quiescent/Low | Weak Repressed PolyComb | Quiescent/Low | Weak Repressed PolyComb | Weak transcription |
| chrX | 45491364 | 45491474 |  |  | Quiescent/Low | Quiescent/Low | Weak Repressed PolyComb | Quiescent/Low | Quiescent/Low | Quiescent/Low |
| chrX | 53600877 | 53600960 |  |  | Strong transcription | Strong transcription | Strong transcription | Strong transcription | Strong transcription | Quiescent/Low |
| chrX | 62922606 | 62922692 |  |  | Quiescent/Low | Strong transcription | Weak transcription | Strong transcription | Weak transcription | Quiescent/Low |
| chrX | 73423663 | 73423769 |  |  | Weak transcription | Strong transcription | Strong transcription | Strong transcription | Quiescent/Low | Quiescent/Low |
| chrX | 73423845 | 73423917 |  |  | Weak transcription | Strong transcription | Strong transcription | Strong transcription | Quiescent/Low | Quiescent/Low |
| chrX | 76142219 | 76142317 |  |  | Quiescent/Low | Quiescent/Low | Weak Repressed PolyComb | Quiescent/Low | Quiescent/Low | Quiescent/Low |
| chrX | 113903999 | 113904079 |  |  | Quiescent/Low | Weak Repressed PolyComb | Weak Repressed PolyComb | Quiescent/Low | Quiescent/Low | Quiescent/Low |
| chrX | 113964272 | 113964383 |  |  | Quiescent/Low | Quiescent/Low | Weak Repressed PolyComb | Quiescent/Low | Quiescent/Low | Quiescent/Low |
| chrX | 133131073 | 133131148 |  |  | Enhancers | Quiescent/Low | Weak Repressed PolyComb | Quiescent/Low | Quiescent/Low | Quiescent/Low |
| chrX | 133131736 | 133131807 |  |  | Enhancers | Quiescent/Low | Weak Repressed PolyComb | Quiescent/Low | Quiescent/Low | Quiescent/Low |
| chrX | 133131893 | 133131974 |  |  | Enhancers | Quiescent/Low | Weak Repressed PolyComb | Quiescent/Low | Quiescent/Low | Quiescent/Low |
| chrX | 133501880 | 133501958 |  |  | Quiescent/Low | Quiescent/Low | Quiescent/Low | Quiescent/Low | Quiescent/Low | Quiescent/Low |
| chrX | 133503036 | 133503133 |  |  | Quiescent/Low | Quiescent/Low | Weak Repressed PolyComb | Quiescent/Low | Quiescent/Low | Quiescent/Low |
| chrX | 133508023 | 133508094 |  |  | Active TSS | Active TSS | Active TSS | Active TSS | Active TSS | Active TSS |
| chrX | 133508309 | 133508407 |  |  | Active TSS | Active TSS \| Flanking Active TSS | Active TSS \| Flanking active TSS | Active TSS | Active TSS | Active TSS |
| chrX | 137577537 | 137577620 |  |  | Quiescent/Low | Quiescent/Low | Weak Repressed PolyComb | Quiescent/Low | Quiescent/Low | Quiescent/Low |
| chrX | 138833972 | 138834056 |  |  | Weak transcription | Strong transcription \| Weak transcription | Weak transcription | Weak transcription | Quiescent/Low | Quiescent/Low |
| chrX | 144883484 | 144883561 |  |  | Heterochromatin | Quiescent/Low | Quiescent/Low | Quiescent/Low | Quiescent/Low | Quiescent/Low |
| chrX | 144883993 | 144884070 |  |  | Quiescent/Low | Quiescent/Low | Quiescent/Low | Quiescent/Low | Quiescent/Low | Quiescent/Low |
| chrX | 146120193 | 146120287 |  |  | Quiescent/Low | Quiescent/Low | Quiescent/Low | Quiescent/Low | Quiescent/Low | Quiescent/Low |
| chrX | 146147969 | 146148060 |  |  | Heterochromatin | Quiescent/Low | Quiescent/Low | Quiescent/Low | Quiescent/Low | Quiescent/Low |
| chrX | 150878755 | 150878840 |  |  | Weak Repressed PolyComb | Quiescent/Low | Weak Repressed PolyComb | Weak transcription | Weak transcription | Quiescent/Low |

ARID3A:AT-Rich Interaction Domain 3A, BHLHE40:Basic Helix-Loop-Helix Family Member E40, CEBPB:CCAAT/Enhancer Binding Protein Beta, CEBPD:CCAAT/Enhancer Binding Protein Delta, CTCF:CCCTC-Binding Factor, ELF1:E74 Like ETS Transcription Factor 1, EP300:E1A Binding Protein P300, FOSL2:FOS Like Antigen 2, FOXA1:Forkhead Box A1, FOXA2:Forkhead Box A2, GABPA:GA Binding Protein Transcription Factor Alpha Subunit, HDAC2:Histone Deacetylase 2, HNF4A:Hepatocyte Nuclear Factor 4 Alpha, HNF4G:Hepatocyte Nuclear Factor 4 Gamma, HSF1:Heat Shock Transcription Factor 1, JUN:Jun Proto-Oncogene, JUND:Jun D Proto-Oncogene, MAFF:V-Maf Avian Musculoaponeurotic Fibrosarcoma Oncogene Homolog F, MAFK:V-Maf Avian Musculoaponeurotic Fibrosarcoma Oncogene Homolog K, MAX:MYC Associated Factor X, MAZ:MYC Associated Zinc Finger Protein, MXI1:MAX Interactor 1, Dimerization Protein, MYBL2:MYB Proto-Oncogene Like 2, NFIC:Nuclear Factor I C, NRF1:Nuclear Respiratory Factor 1, PGC1A:PPARG Coactivator 1 Alpha, POLR2A:Polymerase (RNA) II Subunit A, RAD21:RAD21 Cohesin Complex Component, RCOR1:REST Corepressor 1, REST:RE1 Silencing Transcription Factor, RFX5:Regulatory Factor X5, RXRA:Retinoid X Receptor Alpha, SIN3AK2:Paired amphipathic helix protein, SMC3:Structural Maintenance Of Chromosomes 3, SP1:Specificity Protein 1, SREBP1:Sterol regulatory element-binding protein 1, TAF1:TATA-Box Binding Protein Associated Factor 1, TBP:TATA-Box Binding Protein, YY1:YY1 Transcription Factor
