# Supplementary material for: Genetic variation in long noncoding RNAs and the risk of nonalcoholic fatty liver disease
Source: Oncotarget. 2017 Feb 11;8(14):22917–26. doi: 10.18632/oncotarget.15286 (PMC5410273; doi:10.18632/oncotarget.15286)
Supplement: Supplementary file 5 [file oncotarget-08-22917-s005.doc]

**Supplementary Table 6**

Features of SNPs in lncRNAs-regions associated with NAFLD in the exploratory study and further followed-up in the replication stage

| **Features** | **rs2829145** | **rs11171490** |
| --- | --- | --- |
| **Variant features** | | |
| **Alleles** | **G/A**|Ancestral: **G** | **C/T**|Ancestral: **C** |
| **Location** | Chromosome 21:24495924  (forward strand) | Chromosome 12:55480814  (forward strand) |
| **Gene regulation: gene location** | ENSG00000237484 (2809 bp) (AP000476.1: located Chromosome 21: 25,801,054-25,920,256 in GRCh37 coordinates, forward strand) | **ENSG00000258763**(RP11-110A12.2: located Chromosome 12: 55,828,518-55,979,255 in GRCh37 coordinates, reverse strand) |
| **Gene regulation: transcript** | ENST00000415182 (+) Allele A biotype: lincRNA | This variant overlaps 4 transcripts  [ENST00000554049](http://www.ensembl.org/Homo_sapiens/Transcript/Variation_Transcript/Table?db=core;source=dbSNP;t=ENST00000554049;v=rs11171490;vdb=variation;vf=6357743) (-) biotype: antisense (T)  [ENST00000555138](http://www.ensembl.org/Homo_sapiens/Transcript/Variation_Transcript/Table?db=core;source=dbSNP;t=ENST00000555138;v=rs11171490;vdb=variation;vf=6357743) (-) biotype: antisense (T)  [ENST00000556750](http://www.ensembl.org/Homo_sapiens/Transcript/Variation_Transcript/Table?db=core;source=dbSNP;t=ENST00000556750;v=rs11171490;vdb=variation;vf=6357743) (-) biotype: antisense (T)  [ENST00000555146](http://www.ensembl.org/Homo_sapiens/Transcript/Variation_Transcript/Table?db=core;source=dbSNP;t=ENST00000555146;v=rs11171490;vdb=variation;vf=6357743) (-) biotype: antisense (T) |
| **MAF** | 0.18 (A) | 0.07 (T) |
| **lncRNA features** | | |
| **lncRNA transcript ID*** | lnc-JAM2-6:4 | 5 lncRNA transcripts  lnc-OR6C70-1:1  lnc-OR6C70-1:3  lnc-OR6C70-1:4  lnc-OR6C70-1:2  lnc-OR6C70-1:5 |
| **Gene ID name** | lnc-JAM2-6 | lnc-OR6C70-1 |
| **Location (hg19)** | chr21:25801095-25920256 | chr12:55828539-55979255  chr12:55828541-55886963  chr12:55828521-55873256  chr12:55828544-55979255  chr12:55828518-55873256 |
| **Strand** | + | - |
| **Transcript size** | 2176 | 555/517/532/497/535 |
| **Non code transcript ID ¥** | NONHSAT081453.2 | NONHSAT028660.2  NONHSAT028661.2  NONHSAT028662.2  NONHSAT028663.2 |
| **Non code gene ID ¥** | NONHSAG032538.2 | NONHSAG011311.2 |
| **Exon number** | 3 | 3/4/3/3 |
| **Strand** | + | - |
| **Length** | 2176 bp | 1540 |
| **Expression profile #** | WBC: 0.0613624  Adrenal: 0.133666  Kidney: 0.0403967  Colon: 0.0477087  Heart: 0.0713987  Lymp node: 0.132376  Thyroid: 0.0331275 | Heart: 7.48719e-07  WBC: 0.0150824 |
| **Protein interaction##** | **Pumilio2**: Pumilio RNA Binding Family Member 2 (the encoded protein functions as a translational repressor during embryonic development and cell differentiation)  **eIF4AIII**: Eukaryotic Translation Initiation Factor 4A3 (implicated in a number of cellular processes involving alteration of RNA secondary structure, such as translation initiation, nuclear and mitochondrial splicing, and ribosome and spliceosome assembly).  **TNRC6:** Trinucleotide Repeat Containing 6A (the protein functions in post-transcriptional gene silencing through the RNA interference (RNAi) and microRNA pathways. The protein associates with messenger RNAs and Argonaute proteins in cytoplasmic bodies known as GW-bodies or P-bodies.  **FUS**: encodes a multifunctional protein component of the heterogeneous nuclear ribonucleoprotein (hnRNP) complex. | - |
| **Prediction of regulatory elements: potential sequence-specific motifs binding of transcription factors** | MAFK (V-Maf Avian Musculoaponeurotic Fibrosarcoma Oncogene Homolog K), RAD21 (RAD21 Cohesin Complex Component), JUND (Jun D Proto-Oncogene), and CEBPB | ATF4 (activation transcription factor 4, the encoded protein belongs to cAMP-response element binding protein 2 CREB-2)  SPDEF (SAM Pointed Domain Containing ETS Transcription Factor) |
| **Epigenetic marks** ** | H3K4 Histone Methylation Position in Liver Cells  Methylation Position in PBMC Cell line | H3K36 Histone Methylation Position in Liver Cells |
| **Impact on chromatin structure or histone modifications∞** | **Potential chromatin changes (**Znf4c50c4) Potential histone modifications: fetal heart, intestine, muscle and kidney, primary T helper memory cells from peripheral blood and neutrophils, stomach, duodenum and colon mucosa, pancreas and liver | Potential chromatin changes in hepatocytes  Potential histone modifications: colonic and stomach mucosa, fetal heart and kidney, Primary T cells effector/memory enriched from peripheral blood. |
| Target miRNAs **(MirTarget2)**  **ALE-HSA21** | hsa-miR-4772-3p (score: 91.56)/ hsa-miR-3124-3p (score: 89.31)/ hsa-miR-4727-5p (score: 87.64)/ hsa-miR-4795-3p (score: 85.30)/ hsa-miR-892a (score: 83.90)/ hsa-miR-29a-5p (score: 82.41)/ hsa-miR-5190 (score: 80.89)/ hsa-miR-4693-5p (score: 80.51)/ hsa-miR-656 (score: 80.01).  hsa-miR-375/ hsa-miR-19a/ hsa-miR-19b/ | No microRNA targets found |

Data extracted from http://www.ensembl.org/Homo_sapiens/Variation/ Human GR Ch 38 p.7.

*Prediction of lncRNA transcript: http://www.lncipedia.org/db (last version May 2016)

¥ Non code transcript and gene ID: <http://www.noncode.org/>

**#**Expression profile**:** Human body map 2.0 (<http://www.ensembl.info/blog/2011/05/24/human-bodymap-2-0-data-from-illumina/>.

##Prediction of protein interaction: <http://lncrnator.ewha.ac.kr/> and Starbase V2.0 <http://starbase.sysu.edu.cn/>.

** Prediction Methylation & Histone Modifications: http://genome.igib.res.in/lncRNome/

Prediction of miRNA**:** MirTarget2 (The higher the score, the more confidence the prediction; a predicted target with prediction score > 80 is most likely to be real). WBC: white blood cells.

∞ Prediction of regulatory elements in the intergenic regions of the H. sapiens genome:

RegulomeDB available at: <http://www.regulomedb.org/snp/>.

ALE-HSA21 available at: <http://bioinfo.na.iac.cnr.it/> was used for computational analysis of interaction between noncoding transcripts and miRNAs from (AnaLysis of Expression on HSA21). This web resource integrates—for all coding and noncoding transcripts of chromosome 21—existing gene annotations and transcripts identified *de novo* through RNA-Seq analysis with predictive computational analysis of regulatory sequences.
